# Changes in Ponderal Index and Body Mass Index across Childhood and Their Associations with Fat Mass and Cardiovascular Risk Factors at Age 15

**DOI:** 10.1371/journal.pone.0015186

**Published:** 2010-12-08

**Authors:** Laura D. Howe, Kate Tilling, Li Benfield, Jennifer Logue, Naveed Sattar, Andy R. Ness, George Davey Smith, Debbie A. Lawlor

**Affiliations:** 1 MRC Centre for Causal Analyses in Translational Epidemiology, University of Bristol, Bristol, United Kingdom; 2 School of Social and Community Medicine, University of Bristol, Bristol, United Kingdom; 3 Faculty of Medicine, British Heart Foundation (BHF) Glasgow Cardiovascular Research Centre, University of Glasgow, Glasgow, United Kingdom; 4 Department of Oral and Dental Science, University of Bristol, Bristol, United Kingdom; Lerner Research Institute, Cleveland Clinic, United States of America

## Abstract

**Background:**

Little is known about whether associations between childhood adiposity and later adverse cardiovascular health outcomes are driven by tracking of overweight from childhood to adulthood and/or by vascular and metabolic changes from childhood overweight that persist into adulthood. Our objective is to characterise associations between trajectories of adiposity across childhood and a wide range of cardiovascular risk factors measured in adolescence, and explore the extent to which these are mediated by fat mass at age 15.

**Methods and Findings:**

Using data from the Avon Longitudinal Study of Parents and Children, we estimated individual trajectories of ponderal index (PI) from 0–2 years and BMI from 2–10 years using random-effects linear spline models (N = 4601). We explored associations between PI/BMI trajectories and DXA-determined total-body fat-mass and cardiovascular risk factors at 15 years (systolic and diastolic blood pressure, fasting LDL- and HDL-cholesterol, triglycerides, C-reactive protein, glucose, insulin) with and without adjustment for confounders. Changes in PI/BMI during all periods of infancy and childhood were associated with greater DXA-determined fat-mass at age 15. BMI changes in childhood, but not PI changes from 0–2 years, were associated with most cardiovascular risk factors in adolescence; associations tended to be strongest for BMI changes in later childhood (ages 8.5–10), and were largely mediated by fat mass at age 15.

**Conclusion:**

Changes in PI/BMI from 0–10 years were associated with greater fat-mass at age 15. Greater increases in BMI from age 8.5–10 years are most strongly associated with cardiovascular risk factors at age 15, with much of these associations mediated by fat-mass at this age. We found little evidence supporting previous reports that rapid PI changes in infancy are associated with future cardiovascular risk. This study suggests that associations between early overweight and subsequent adverse cardiovascular health are largely due to overweight children tending to remain overweight.

## Introduction

The prevalence of obesity in children and adolescents has risen dramatically in recent decades across most western countries and several low-income countries[1], although some recent data suggest prevalence may have stabilised in the USA, UK and Sweden.[2]–[4] A systematic review demonstrated that high body mass index (BMI) from age seven onwards is associated with an increased risk of coronary heart disease in adulthood.[5] Several cross-sectional studies have demonstrated that childhood obesity is associated with increased levels of cardiovascular risk factors.[6]–[9] These associations may be driven by tracking of overweight from childhood to adulthood [10] and/or by vascular and metabolic changes from childhood overweight that persist into adulthood. Few studies have been able to explore the associations between early childhood adiposity and later cardiovascular risk prospectively, or to explore the extent to which these associations are mediated by the tracking of adiposity.

Changes in adiposity across childhood are also of interest for their association with later obesity itself. A recent small study (N = 233) from a UK cohort showed that most excess weight gain up to age 9 occurs before the age of 5, suggesting that obesity prevention efforts should be concentrated on pre-school children.[11] This study, however, focused on weight gain rather than a measure of adiposity *per se*, did not have data beyond the age of 9, and did not examine the association of childhood weight gain with future cardiovascular risk factors. Other studies, including previous reports using data from the same cohort as we analyse in this study, have suggested that rapid weight gain or gain in BMI or ponderal index (PI) in infancy (i.e. prior to 2 years) is importantly associated with increased obesity and adverse cardiovascular risk factors in later life.[12]–[14] However, few of these studies have been able to compare the associations of changes in weight (or weight adjusted for height) during infancy with later outcomes with the associations of growth in later periods of childhood with these outcomes. Additionally, most previous studies have relied on using repeat z-scores of weight (or weight adjusted for height), rather than modelling growth trajectories, and therefore have incompletely modelled the clustering of measurements within individuals.

In this paper, we model individual trajectories of PI and BMI across childhood from a large UK cohort study using linear spline random effects models. This allowed us to identify distinct periods of PI/BMI change in childhood, and explore the associations of PI/BMI changes in each of these periods with fat mass and cardiovascular risk factors in adolescence. Where associations were found with later cardiovascular risk factors we further aimed to explore the extent to which these were mediated by fat mass at age 15.

## Methods

### Study population

ALSPAC is a prospective cohort study. The full study methodology is published elsewhere[15], and on the study website (www.bristol.ac.uk/alspac). Pregnant women resident in one of three Bristol-based health districts with an expected delivery date between 1 April 1991 and 31 December 1992 were invited to participate. Of these women, 14,541 were recruited; there were 14,062 live-born children, 13,988 of whom were alive at one year. Follow-up has included parent- and child-completed questionnaires, links to routine data, and clinic attendance. Ethical approval for the study was obtained from the ALSPAC Law and Ethics Committee and the Local Research Ethics Committees; written consent was obtained from all participants and their parents/guardians.

### Measurements

Within ALSPAC, the only measures of adiposity with repeat measures across the whole of childhood are those based on height and weight. Length/height and weight data for the children are available from several sources. Birth length (crown-heel) was measured by ALSPAC staff who visited newborns soon after birth (median 1 day, range 1-14 days), using a Harpenden Neonatometer (Holtain Ltd). Birth weight was extracted from medical records. From birth to five years, length and weight measurements were extracted from health visitor records, which form part of standard child care in the UK. In this cohort we had up to four measurements taken on average at six weeks, 10, 21, and 48 months of age, which previous work has shown to have good accuracy.[16] For a random 10% of the cohort, we also have length/height and weight measurements from research clinics, held between the ages of four months and five years of age. From age seven years upwards, all children were invited to annual clinics. Details of measuring equipment used in the clinics are in **Supporting File S1**. Across all ages, parent-reported child heights and weights are also available from questionnaires. PI was calculated as weight in kilograms divided by height in metres cubed; BMI was calculated as weight in kilograms divided by height in metres squared.

The outcomes in these analyses are from the research clinic held at approximately age 15 (mean age 15.5 years). Total body fat mass was assessed by dual X-ray absorptiometry (DXA) scan using a Lunar prodigy narrow fan beam densitometer. The cardiovascular risk factors we examined were systolic and diastolic blood pressure and fasting blood measures (LDLc, HDLc, triglycerides, CRP, glucose and insulin), measured using standard procedures detailed in the **Supporting File S2.**

The following were considered as potential confounders related both to childhood PI/BMI trajectories and adolescent fat mass or cardiovascular risk factors: gender, maternal and partner education, household social class, maternal age, height, gestational age at birth, maternal and partner BMI, maternal and partner smoking in pregnancy, age at clinic attendance, and pubertal stage at measurement of outcome. Details of measurement of confounding factors are presented in the **Supporting File S3**.

### PI/BMI trajectories across childhood

BMI (kg/m^2^) is the most common way of adjusting weight for height in adults to obtain a measure of adiposity free from the influence of height. Patterns of BMI change in early childhood are extremely complicated. Given this, we decided not to model BMI from birth. Rather, PI (kg/m^3^) was used as the measure of adiposity from birth to two years. BMI was modelled from two to ten years. Implausible height and weight measurements (>4 SD from the mean for gender and age specific category, approximately 0.1% of all measurements) were re-coded as missing. All other available measures were used in analyses. To account for the likely reduced accuracy of parent-reported measurements[17], a binary indicator of measurement source (research clinic or health records versus parent-report) was included in all models.

Individual trajectories of PI/BMI were estimated using mixed-effects linear spline models (two levels: measurement occasion and individual), fitted using the statistical package MLwiN version 2.10 (www.cmm.bristol.ac.uk/MLwiN/index.shtml). Such models allow for the change in scale and variance of PI/BMI over time and use all available data from all eligible children under a missing at random assumption. They allow for individual variation in trajectories, since random effects allow each individual to have different intercepts and slopes (rates of PI/BMI change). Trajectories were modelled separately for boys and girls. BMI trajectories were not modelled beyond age ten since puberty would necessitate individual spline points due to variation in age at puberty onset. Modelling PI/BMI to age 10 also allows clear separation in time (age) between the exposure (trajectories in childhood to age 10) and outcomes (fat mass and cardiovascular risk factors assessed at mean age 15) assessed in this prospective study and thus make reverse causality extremely unlikely. Full details of the statistical methodology are in the **Supporting File S4**.

### Statistical analysis of associations between PI/BMI trajectories and outcomes at age 15

Analyses were restricted to singletons with at least two PI measures between 0–2 years and two BMI measures between 2–10 years, who attended the 15 year follow-up clinic. The associations between PI/BMI trajectories and fat mass and cardiovascular risk factors were modelled using linear regressions in Stata 11.[18]

We present the associations between each period of PI/BMI change and each outcome adjusted for:

- Age (Model 1)
- Age and previous periods of PI/BMI change (Model 2)
- Age, previous periods of PI/BMI change and potential confounders (Model 3, see above for list of confounders)

To explore the extent to which associations between PI/BMI changes during childhood and later outcomes are mediated by adiposity at the time of outcome measurement, we further adjusted associations with cardiovascular risk factors for DXA-assessed total fat mass, height, and height squared measured at age 15 (*Model 4*). Adjusting for height and height squared is necessary to obtain a measure of fat mass at age 15 that is independent of height. The models adjusted for fat mass at age 15 (Model 4) show whether there are associations between childhood PI/BMI changes and cardiovascular risk factors at age 15 independent of adiposity at the time of measurement of the cardiovascular risk factor.

We used multivariate multiple imputation to impute missing variables, including all covariables and potential predictors in the imputation equations; details of the procedure are presented in the **Supporting File S5**. Analyses limited to individuals with complete data for all variables were also conducted; the findings did not differ from the imputed data results (available from the authors on request).

There was evidence of interaction between gender and PI/BMI trajectories for DXA-assessed fat mass, and for some, but not all, cardiovascular risk factors; for consistency all results are presented separately for boys and girls.

DXA-assessed total fat mass, triglycerides, CRP and insulin were all right-skewed and so were analysed on the natural log scale. All exposure, outcome and confounder variables were standardised (subtracted the mean, divided by the standard deviation) prior to analysis. Thus associations represent the standard deviation change in outcome that is observed with a one standard deviation increase in the rate of PI/BMI change, presented with 95% confidence intervals. For some periods of PI/BMI change in childhood, PI/BMI is decreasing on average; these periods are shaded in the tables. In these periods, a positive coefficient for the relationship between PI/BMI change and the outcome means that a shallower negative gradient, i.e. slower rate of PI/BMI decrease, is associated with a higher level of outcome, or vice versa for a negative coefficient.

## Results

### Data and population

5113 singletons attended the 15-year research clinic, approximately 50% of those invited. Of these, PI/BMI trajectories were available for 4601 (90.0%). These 4601 individuals tended to have higher maternal education, older maternal age, and higher birth weight compared with the full ALSPAC cohort, but no differences in maternal BMI were observed (**Table S1**). **Table 1** describes the participants, their fat mass and cardiovascular risk factors, and levels of missing data.

**Table 1:** Summary of 5113 participants attending the 15-year research clinic, and their body composition and cardiovascular risk factor measurements.

		N (%) with missing data	Boys	Girls	P value for sex interaction between BMI trajectories and outcomes
			*N = 2413*	*N = 2700*	
Birth weight (kg)	Mean (SD)	68	3.49 (0.49)	3.39 (0.49)	
Gestational age (complete weeks)	Mean (SD)	0	39.40 (1.87)	39.60 (1.68)	
Age at clinic attendance (months)	Mean (SD)	0	185.39 (3.87)	185.86 (4.55)	
Puberty (Tanner stage)	N (%)	659			
*Stage 1/2*			21 (1.0)	7 (0.3)	
*Stage 3*			136 (6.7)	130 (5.6)	
*Stage 4*			1360 (66.7)	1635 (69.8)	
*Stage 5*			521 (25.6)	569 (24.3)	
BMI at clinic attendance (kg/m^2^)	Median (IQR)	297	21.01 (3.84)	21.81 (3.84)	
DXA-assessed fat mass (kg)	Median (IQR)	336	8.60 (5.87 to 13.74)	17.37 (13.32 to 22.52)	<0.001
Systolic blood pressure (mmHg)	Mean (SD)	390	125.97 (10.36)	120.56 (10.49)	0.91
Diastolic blood pressure (mmHg)	Mean (SD)	390	68.29 (9.11)	66.97 (8.33)	0.53
Fasting LDL cholesterol (mmol/l)	Mean (SD)	1865	1.99 (0.52)	2.18 (0.57)	0.59
Fasting HDL cholesterol (mmol/l)	Mean (SD)	1865	1.21 (0.27)	1.35 (0.30)	0.77
Fasting triglycerides (mmol/l)	Median (IQR)	1865	0.73 (0.57 to 0.97)	0.77 (0.62 to 1.00)	0.38
Fasting glucose (mmol/l)	Mean (SD)	1865	5.30 (0.39)	5.14 (0.38)	0.001
Fasting insulin (IU/l)	Median (IQR)	1869	8.20 (6.01 to 11.16)	9.87 (7.41 to 13.15)	0.26
C-reactive protein (mg/l)	Median (IQR)	1865	0.38 (0.22 to 0.90)	0.40 (0.22 to 0.90)	0.17

### PI/BMI trajectories

**Figure 1:**
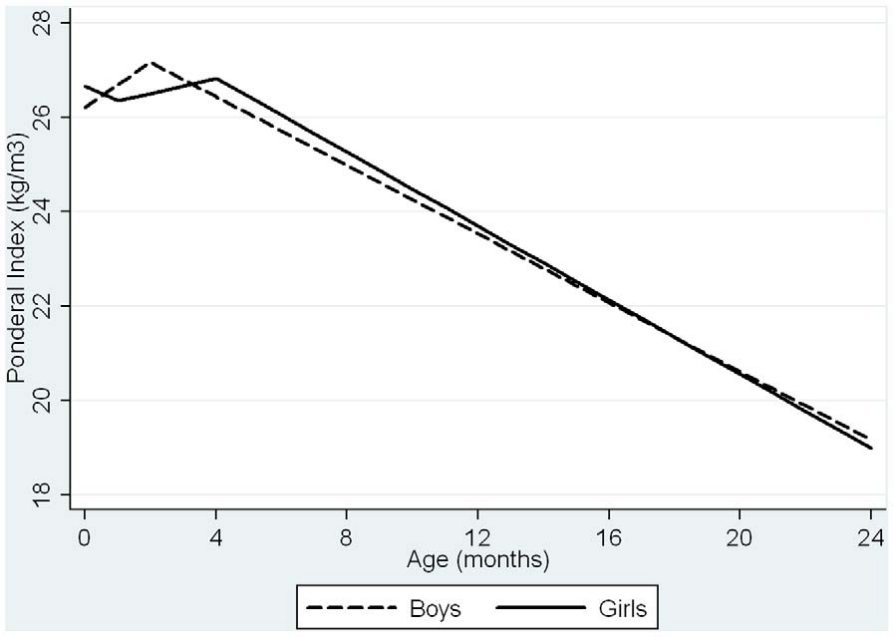
Boys Overall PI/BMI trajectories predicted by random effects models.

The random effects linear spline modelling confirmed that there were 2 periods of PI change for boys (0–2 months, 2–24 months), 3 periods of PI change for girls (0–1 month, 1–4 months, 4–24 months), and 6 periods of BMI change for boys (24–60 months, 60–65 months, 65–75 months, 75–81 months, 81–103 months, 103–120 months) and girls (24–56 months, 56–67 months, 67–73 months, 73–79 months, 79–105 months, 105–120 months) (**Figures 1** **&** **2** & **Table S2**). For simplicity, we refer to the BMI change periods as the approximate ages in years, i.e. change periods are 2–5, 5–5.5, 5.5–6.5, 6.5–7, 7–8.5 and 8.5–10 years.

**Figure 2:**
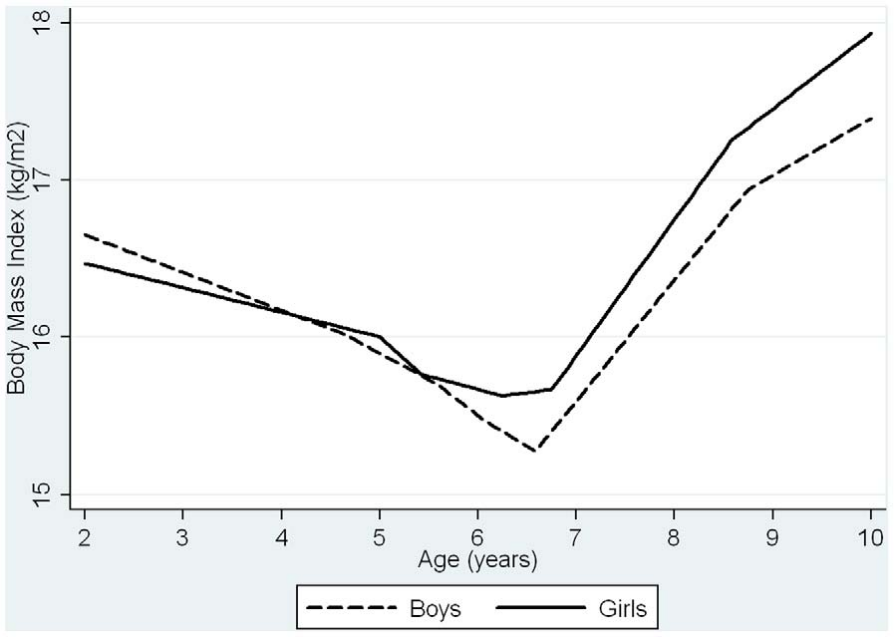
Girls Overall PI/BMI trajectories predicted by random effects models.

### Childhood PI/BMI trajectories and DXA-assessed total body fat mass at age 15

**Table S3** presents results from all three models (Model 1: age adjusted; Model 2: age and growth in previous period adjusted; Model 3: age, growth in previous period and other confounder adjusted). The effect of adjustment for previous periods of PI/BMI change appears to be complex (Model 2, **Table S3**), with adjustment resulting in strengthened associations for some periods of PI/BMI change, and attenuated associations for others. Further adjustment for confounders has relatively little effect on the associations (Model 3, **Table S3**).

**Figure 3:**
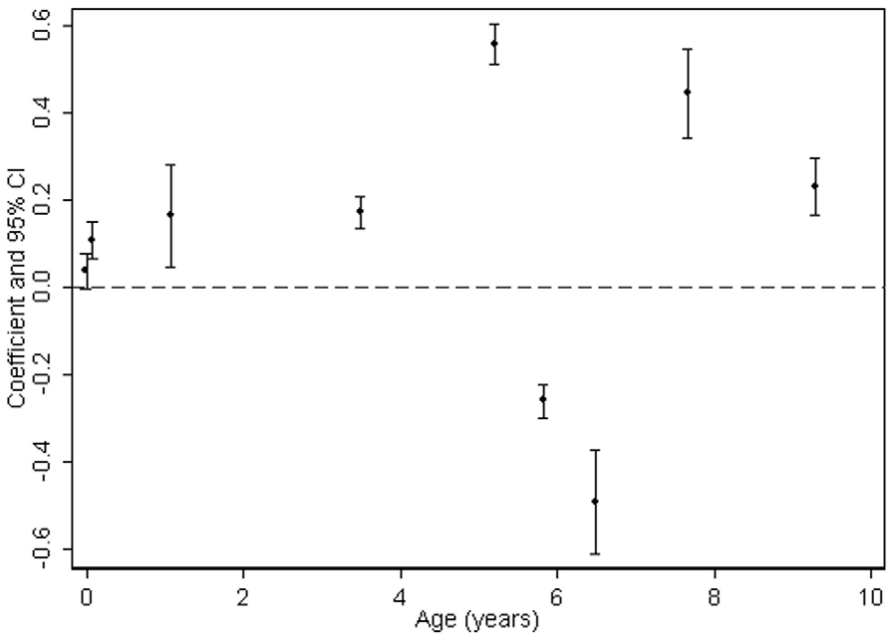
Boys Coefficients and 95% confidence intervals from standardised linear regressions of DXA-assessed fat mass at age 15 on PI/BMI trajectories (age in years along x-axis is the mid-point of PI/BMI change periods; coefficients are adjusted for confounders and previous PI/BMI changes, i.e. Model 3).

**Table 2** presents only the full confounder (including growth in previous period) model (equivalent to Model 3 in web-table 4) and **Figures 3** **and** **4** also show these associations and allows easy comparison of strengths of association for different PI/BMI change periods. In these fully adjusted models, PI at birth and all periods of PI/BMI change were associated with DXA-assessed total fat mass at age 15 (**Table 2** and **Figures 3** **&** **4**). For most periods of PI/BMI change, the associations were positive such that faster rates of PI/BMI increase (or slower rates of PI/BMI decline in periods where PI/BMI is decreasing) were associated with higher fat mass at age 15 (**Table 2**). Between 5.5–6.5 and 6.5–7 years, coefficients for the association between BMI change and fat mass at age 15 were negative. BMI is on average declining across this period (**Figures 1** **&** **2**/**Table S2**); a faster rate of BMI decrease is therefore associated with increased fat mass at age 15. These negative associations may reflect timing of adiposity rebound, i.e. those with earlier adiposity rebound may have faster rates of BMI decrease in these periods and higher fat mass later.[19]

**Figure 4:**
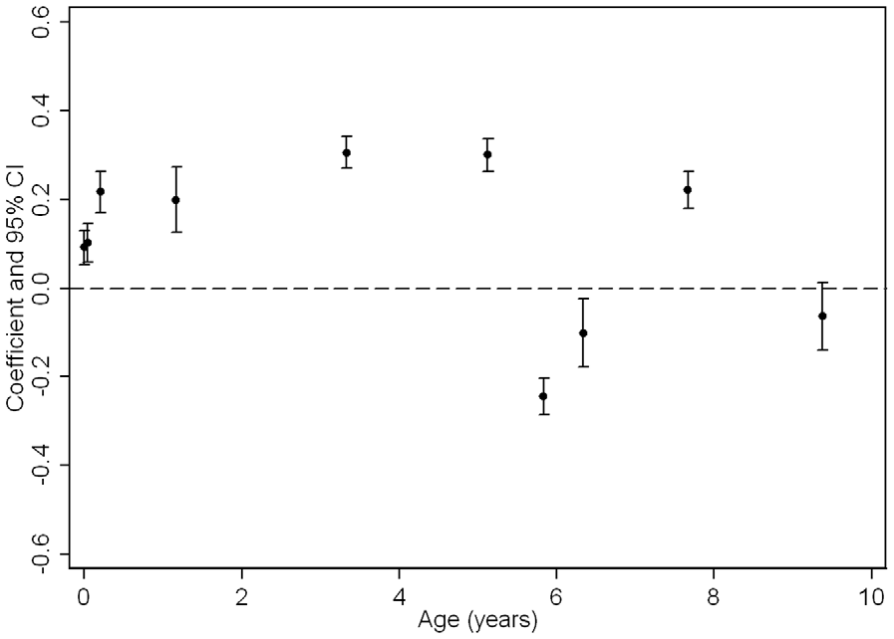
Girls Coefficients and 95% confidence intervals from standardised linear regressions of DXA-assessed fat mass at age 15 on PI/BMI trajectories (age in years along x-axis is the mid-point of PI/BMI change periods; coefficients are adjusted for confounders and previous PI/BMI changes, i.e. Model 3).

**Table 2:** PI/BMI trajectories from birth to ten years and their association with Ln of DXA-assessed total body fat mass at age 15 years, with multiple imputation, adjusted for age, previous periods of PI/BMI change and confounders.

PI/BMI change period	Logged DXA-assessed Fat Mass
	Model 3
*Boys, N = 2181*	
PI at birth	0.038 (−0.004,0.079)
PI change 0–2mt	0.109 (0.067,0.152)
**PI change 2–24mt**	**0.164 (0.048,0.281)**
**BMI change 2–5y**	**0.172 (0.134,0.209)**
**BMI change 5–5.5y**	**0.558 (0.511,0.605)**
**BMI change 5.5–6.5y**	**−0.259 (−0.298,−0.220)**
**BMI change 6.5–7y**	**−0.491 (−0.612, −0.370)**
BMI change 7–8.5y	0.446 (0.343,0.549)
BMI change 8.5–10y	0.232 (0.165,0.299)
*Girls, N = 2420*	
PI at birth	0.093 (0.054,0.131)
**PI change 0–1 m**	**0.102 (0.058,0.146)**
PI change 1–4 m	0.218 (0.171,0.264)
**PI change 4–24 m**	**0.200 (0.126,0.273)**
**BMI change 2–5y**	**0.306 (0.271,0.342)**
**BMI change 5–5.5y**	**0.301 (0.264,0.337)**
**BMI change 5.5–6.5y**	**−0.244 (−0.286,−0.203)**
BMI change 6.5–7y	−0.101 (−0.178, −0.024)
BMI change 7–8.5y	0.222 (0.180,0.263)
BMI change 8.5–10y	−0.063 (−0.139,0.012)

All variables were standardised prior to analysis. Coefficients represent the standard deviation change in DXA-assessed total body fat mass associated with a one standard deviation increase in the rate of PI/BMI change.

**Bold text** indicates that PI/BMI is, on average, declining during that period.

Adjusted for age, previous periods of PI/BMI change, height, height squared, gender, maternal and partner education, household social class, maternal age, height, gestational age at birth, maternal and partner BMI, maternal and partner smoking in pregnancy, age at clinic attendance, and pubertal stage at measurement of outcome.

PI/BMI change periods:

BMI change 2–5y: 24 and 60 months for boys, 24 and 56 months for girls.

BMI change 5–5.5y: 60 and 65 months for boys, 56 and 67 months for girls.

BMI change 5.5–6.5y: 65 and 75 months for boys, 67 and 73 months for girls.

BMI change 6.5–7y: 75 and 81 months for boys, 73 and 79 months for girls.

BMI change 7–8.5y: 81 and 103 months for boys, 79 and 105 months for girls.

BMI change 8.5–10y: 103 and 120 months for boys, 105 and 120 months for girls.

### Childhood PI/BMI trajectories and cardiovascular risk factors at age 15

**Tables S4, S5, S6, S7, S8, S9, S10 & S11** present results from all three models for each cardiovascular risk factor (Model 1: age adjusted; Model 2: age and growth in previous period adjusted; Model 3: age, growth in previous period and other confounder adjusted; Model 4: age, growth in previous period, other confounders and fat mass, height and height-squared at age 15). Adjustment for previous periods of PI/BMI change attenuates the associations of some PI/BMI change periods with the cardiovascular risk factors and strengthens the associations of others, and further adjustment for confounders tends to attenuate associations slightly (Models 2 and 3, **Tables S4, S5, S6, S7, S8, S9, S10 & S11**).

**Figure 5:**
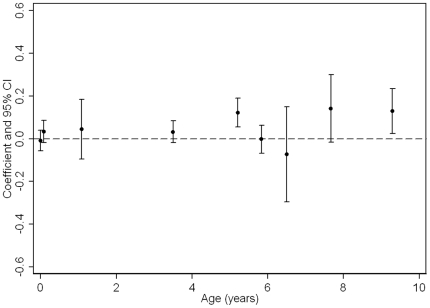
Boys Coefficients and 95% confidence intervals from standardised linear regressions of log triglycerides at age 15 on PI/BMI trajectories (age in years along x-axis is the mid-point of PI/BMI change periods; coefficients are adjusted for confounders and previous PI/BMI changes, i.e. Model 3 in Tables).

In Model 3, looking at the total association of each period of PI/BMI change with the cardiovascular risk factors (adjusted for confounders and previous periods of PI/BMI change), there is very little evidence of associations between PI changes from 0–2 years and cardiovascular risk factors at age 15 (**Table 3**, **Figures 5** **&** **6** for triglycerides, and **Supporting Figures S1, S2, S3, S4, S5, S6, S7, S8, S9, S10, S11, S12, S13 & S14** for the other cardiovascular risk factors). Some periods of BMI change between 2–10 years, however, were associated with several cardiovascular risk factors; in particular BMI changes between 5–5.5 years, 7–8.5 and 8.5–10 years demonstrated associations with several risk factors.

**Figure 6:**
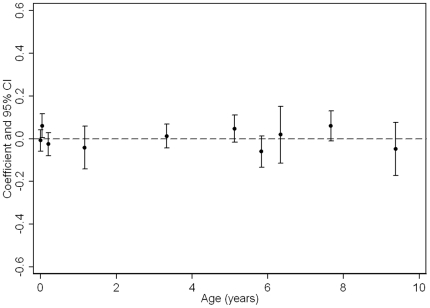
Girls Coefficients and 95% confidence intervals from standardised linear regressions of log triglycerides at age 15 on PI/BMI trajectories (age in years along x-axis is the mid-point of PI/BMI change periods; coefficients are adjusted for confounders and previous PI/BMI changes, i.e. Model 3 in Tables).

**Table 3:** PI/BMI trajectories from birth to ten years and their association with cardiovascular risk factors at age 15, adjusted for previous periods of PI/BMI change and confounders (Model 3).

PI/BMI change period	Systolic blood pressure	Diastolic blood pressure	LDLc	HDLc	Ln triglycerides	Ln CRP	Ln insulin	Glucose
*Boys, N = 2181*								
PI at birth	−0.062	−0.026	0.013	−0.012	−0.009	−0.026	−0.018	−0.028
	(−0.106, −0.018)	(−0.070,0.019)	(−0.040,0.064)	(−0.065,0.042)	(−0.057,0.039)	(−0.080,0.029)	(−0.071,0.035)	(−0.078,0.022)
PI change	0.049	0.034	0.027	−0.062	0.034	0.042	−0.040	−0.038
0–2mt	(0.004,0.095)	(−0.012,0.081)	(−0.034,0.087)	(−0.115, −0.008)	(−0.018,0.085)	(−0.011,0.095)	(−0.098,0.017)	(−0.094,0.019)
**PI change**	**0.053**	**0.032**	**0.062**	**−0.063**	**0.044**	**−0.028**	**−0.055**	**−0.015**
**2–24mt**	**(−0.076,0.182)**	**(−0.096,0.160)**	**(−0.081,0.205)**	**(−0.222,0.096)**	**(−0.095,0.184)**	**(−0.194,0.139)**	**(−0.201,0.092)**	**(−0.159,0.130)**
**BMI change**	**0.014**	**−0.001**	**0.018**	**−0.045**	**0.032**	**0.025**	**0.053**	**−0.001**
**2–5y**	**(−0.028,0.056)**	**(−0.044,0.042)**	**(−0.036,0.072)**	**(−0.104,0.013)**	**(−0.019,0.083)**	**(−0.030,0.081)**	**(−0.001,0.106)**	**(−0.061,0.060)**
**BMI change**	**0.012**	**−0.013**	**0.112**	**−0.129**	**0.122**	**0.211**	**0.226**	**0.040**
**5–5.5y**	**(−0.047,0.071)**	**(−0.073,0.047)**	**(0.033,0.191)**	**(−0.205, −0.053)**	**(0.055,0.189)**	**(0.139,0.283)**	**(0.158,0.294)**	**(−0.035,0.114)**
**BMI change**	**−0.079**	**−0.004**	**−0.046**	**0.040**	**−0.002**	**−0.096**	**−0.089**	**0.015**
**5.5–6.5y**	**(−0.129, −0.028)**	**(−0.057,0.048)**	**(−0.107,0.014)**	**(−0.020,0.099)**	**(−0.068,0.063)**	**(−0.163, −0.028)**	**(−0.154, −0.024)**	**(−0.040,0.070)**
**BMI change**	**0.086**	**0.030**	**−0.247**	**0.078**	**−0.073**	**−0.119**	**−0.156**	**−0.063**
**6.5–7y**	**(−0.086,0.258)**	**(−0.138,0.180)**	**(−0.447, −0.046)**	**(−0.107,0.264)**	**(−0.295,0.150)**	**(−0.319,0.082)**	**(−0.333,0.021)**	**(−0.241,0,115)**
BMI change	0.103	−0.012	0.068	−0.050	0.142	−0.009	0.351	0.103
7–8.5y	(−0.037,0.243)	(−0.153,0.129)	(−0.081,0.217)	(−0.203,0.103)	(−0.016,0.299)	(−0.190,0.171)	(0.184,0.519)	(−0.055,0.261)
BMI change	0.026	0.066	−0.016	−0.128	0.130	0.167	0.090	0.068
8.5–10y	(−0.065,0.117)	(−0.028,0.161)	(−0.121,0.088)	(−0.235, −0.020)	(0.025,0.234)	(0.061,0.273)	(−0.015,0.195)	(−0.032,0.168)
*Girls, N = 2420*								
PI at birth	−0.014	−0.027	0.013	0.031	−0.008	0.014	−0.002	0.010
	(−0.057,0.029)	(−0.070,0.016)	(−0.041,0.067)	(−0.023,0.086)	(−0.058,0.041)	(−0.037,0.064)	(−0.061,0.056)	(−0.042,0.062)
**PI change**	**0.036**	**0.024**	**0.041**	**−0.090**	**0.060**	**0.040**	**0.002**	**0.032**
**0–1m**	**(−0.011,0.083)**	**(−0.022,0.070)**	**(−0.012,0.093)**	**(−0.148, −0.032)**	**(0.005,0.116)**	**(−0.013,0.093)**	**(−0.054,0.058)**	**(−0.019,0.083)**
PI change	0.013	0.013	0.023	0.027	−0.025	0.089	0.007	−0.015
1–4m	(−0.037,0.063)	(−0.038,0.064)	(−0.055,0.101)	(−0.029,0.084)	(−0.080,0.029)	(0.014,0.163)	(−0.057,0.072)	(−0.083,0.052)
**PI change**	**0.045**	**0.021**	**0.015**	**−0.055**	**−0.041**	**0.063**	**0.063**	**0.029**
**4–24m**	**(−0.040,0.131)**	**(−0.064,0.105)**	**(−0.074,0.104)**	**(−0.143,0.033)**	**(−0.141,0.058)**	**(−0.043,0.168)**	**(−0.034,0.159)**	**(−0.061,0.119)**
**BMI change**	**0.054**	**−0.014**	**0.037**	**−0.061**	**0.012**	**0.127**	**−0.007**	**−0.019**
**2–5y**	**(0.010,0.099)**	**(−0.059,0.032)**	**(−0.018,0.093)**	**(−0.112, −0.010)**	**(−0.043,0.068)**	**(0.061,0.193)**	**(−0.061,0.048)**	**(−0.073,0.034)**
**BMI change**	**0.098**	**0.015**	**0.015**	**−0.001**	**0.047**	**0.116**	**0.106**	**−0.008**
**5–5.5y**	**(0.049,0.146)**	**(−0.036,0.065)**	**(−0.048,0.079)**	**(−0.054,0.053)**	**(−0.017,0.111)**	**(0.062,0.170)**	**(0.025,0.186)**	**(−0.084,0.068)**
**BMI change**	**−0.062**	**−0.002**	**−0.057**	**0.032**	**−0.060**	**−0.089**	**−0.068**	**−0.020**
**5.5–6.5y**	**(−0.116, −0.007)**	**(−0.057,0.054)**	**(−0.120,0.005)**	**(−0.043,0.107)**	**(−0.134,0.013)**	**(−0.151, −0.026)**	**(−0.138,0.001)**	**(−0.090,0.050)**
BMI change	−0.029	−0.077	−0.042	−0.075	0.019	−0.014	0.019	0.035
6.5–7y	(−0.132,0.075)	(−0.181,0.028)	(−0.163,0.080)	(−0.207,0.058)	(−0.115,0.152)	(−0.130,0.103)	(−0.099,0.138)	(−0.077,0.147)
BMI change	0.081	0.009	0.060	−0.038	0.060	0.057	0.137	0.053
7–8.5y	(0.023,0.139)	(−0.049,0.068)	(−0.012,0.132)	(−0.117,0.041)	(−0.010,0.130)	(−0.010,0.123)	(0.061,0.214)	(−0.037,0.142)
BMI change	−0.016	0.039	−0.076	0.011	−0.048	−0.042	0.065	0.011
8.5–10y	(−0.122,0.090)	(−0.065,0.143)	(−0.191,0.038)	(−0.100,0.122)	(−0.172,0.076)	(−0.192,0.109)	(−0.041,0.170)	(−0.099,0.120)

All variables were standardised prior to analysis. Coefficients represent the standard deviation change in the cardiovascular risk factor associated with a one standard deviation increase in the rate of PI/BMI change.

**Bold text** indicates that PI/BMI is, on average, declining during that period.

Adjusted for age, previous periods of PI/BMI change, gender, maternal and partner education, household social class, maternal age, height, gestational age at birth, maternal and partner BMI, maternal and partner smoking in pregnancy, age at clinic attendance, and pubertal stage at measurement of outcome.

PI/BMI change periods:

BMI change 2–5y: 24 and 60 months for boys, 24 and 56 months for girls.

BMI change 5–5.5y: 60 and 65 months for boys, 56 and 67 months for girls.

BMI change 5.5–6.5y: 65 and 75 months for boys, 67 and 73 months for girls.

BMI change 6.5–7y: 75 and 81 months for boys, 73 and 79 months for girls.

BMI change 7–8.5y: 81 and 103 months for boys, 79 and 105 months for girls.

BMI change 8.5–10y: 103 and 120 months for boys, 105 and 120 months for girls.

All of the cardiovascular risk factors apart from DBP were strongly associated with DXA-assessed fat mass at age 15 (**Table S12**). In almost all cases, the observed associations between BMI changes between ages 2–10 years and cardiovascular and metabolic risk factors (Model 3, **Table 3**) were attenuated by adjustment for DXA-assessed fat mass, height and height squared at age 15 (Model 4, **Table 4**).

**Table 4:** Adiposity trajectories from birth to ten years and their association with cardiovascular risk factors at age 15, adjusted for previous periods of PI/BMI change, confounders, DXA-assessed fat mass, height and height squared at age 15 (Model 4).

PI/BMI change period	Systolic blood pressure	Diastolic blood pressure	LDLc	HDLc	Ln triglycerides	Ln CRP	Ln insulin	Glucose
*Boys, N = 2181*								
PI at birth	−0.062	−0.021	0.004	−0.005	−0.018	−0.038	−0.034	−0.033
	(−0.105, −0.019)	(−0.065,0.023)	(−0.045,0.054)	(−0.057,0.047)	(−0.065,0.028)	(−0.91,0.015)	(−0.084,0.016)	(−0.083,0.017)
PI change	0.020	0.023	0.020	−0.029	0.009	0.011	−0.092	−0.052
0–2mt	(−0.026,0.065)	(−0.024,0.070)	(−0.042,0,082)	(−0.083,0.025)	(−0.042,0.060)	(−0.043,0.065)	(−0.146,0.038)	(−0.109,0.004)
**PI change**	**0.040**	**0.040**	**0.031**	**−0.030**	**0.006**	**−0.080**	**−0.125**	**−0.037**
**2–24mt**	**(−0.088,0.167)**	**(−0.088,0.167)**	**(−0.109,0.171)**	**(−0.187,0.127)**	**(−0.130,0.142)**	**(−0.241,0.082)**	**(−0.270,0.020)**	**(−0.179,0.105)**
**BMI change**	**0.005**	**0.013**	**−0.020**	**−0.016**	**−0.011**	**−0.032**	**−0.022**	**−0.026**
**2–5y**	**(−0.038,0.048)**	**(−0.031,0.057)**	**(−0.075,0.035)**	**(−0.074,0.043)**	**(−0.062,0.039)**	**(−0.084,0.021)**	**(−0.073,0.030)**	**(−0.088,0.037)**
**BMI change**	**−0.031**	**0.038**	**−0.010**	**−0.034**	**−0.022**	**0.034**	**−0.023**	**−0.055**
**5–5.5y**	**(−0.097,0.035)**	**(−0.028,0.105)**	**(−0.101,0.081)**	**(−0.125,0.058)**	**(−0.106,0.062)**	**(−0.053,0.121)**	**(−0.096,0.050)**	**(−0.141,0.031)**
**BMI change**	**−0.065**	**−0.029**	**0.011**	**−0.005**	**0.069**	**−0.015**	**0.028**	**−0.063**
**5.5–6.5y**	**(−0.119, −0.012)**	**(−0.084,0.026)**	**(−0.048,0.070)**	**(−0.068,0.057)**	**(0.001,0.138)**	**(−0.079,0.048)**	**(−0.035,0.091)**	**(0.006,0.120)**
**BMI change**	**0.130**	**0.013**	**−0.163**	**−0.038**	**0.054**	**0.024**	**0.070**	**0.019**
**6.5–7y**	**(−0.044,0.304)**	**(−0.156,0.182)**	**(−0.359,0.034)**	**(−0.215,0.138)**	**(−0.161,0.269)**	**(−0.177,0.226)**	**(−0.104,0.244)**	**(−0.162,0.199)**
BMI change	0.074	0.023	−0.021	0.039	0.021	−0.151	0.151	0.023
7–8.5y	(−0.068,0.217)	(−0.119,0.165)	(−0.173,0.130)	(−0.119,0.196)	(−0.143,0.184)	(−0.326,0.024)	(−0.013,0.316)	(−0.142,0.189)
BMI change	0.023	0.084	−0.069	−0.090	0.070	0.096	−0.009	0.028
8.5–10y	(−0.069,0.115)	(−0.011,0.179)	(−0.174,0.036)	(−0.198,0.019)	(−0.032,0.172)	(−0.008,0.199)	(−0.115,0.096)	(−0.076,0.133)
*Girls, N = 2420*								
PI at birth	−0.037	−0.032	0.001	0.049	−0.020	−0.015	−0.030	0.007
	(−0.079,0.007)	(−0.076,0.011)	(−0.054,0.055)	(−0.004,0.103)	(−0.068,0.029)	(−0.065,0.034)	(−0.087,0.027)	(−0.045,0.058)
**PI change**	**0.004**	**0.006**	**0.040**	**−0.070**	**0.056**	**0.022**	**−0.034**	**0.028**
**0–1m**	**(−0.043,0.051)**	**(−0.040,0.053)**	**(−0.015,0.096)**	**(−0.126, −0.014)**	**(−0.002,0.114)**	**(−0.027,0.072)**	**(−0.090,0.022)**	**(−0.023,0.078)**
PI change	−0.038	−0.003	−0.003	0.073	−0.050	0.029	−0.063	−0.024
1–4m	(−0.089,0.013)	(−0.055,0.048)	(−0.084,0.078)	(0.017,0.128)	(−0.108,0.008)	(−0.045,0.104)	(−0.126,−0.001)	(−0.090,0.042)
**PI change**	**0.005**	**0.008**	**−0.014**	**−0.014**	**−0.066**	**0.007**	**0.002**	**0.026**
**4–24m**	**(−0.080,0.090)**	**(−0.077,0.093)**	**(−0.104,0.076)**	**(−0.102,0.073)**	**(−0.168,0.035)**	**(−0.097,0.112)**	**(−0.096,0.100)**	**(−0.066,0.119)**
**BMI change**	**−0.008**	**−0.024**	**−0.022**	**−0.001**	**−0.044**	**0.030**	**−0.112**	**−0.031**
**2–5y**	**(−0.056,0.039)**	**(−0.074,0.026)**	**(−0.082,0.037)**	**(−0.058,0.057)**	**(−0.101,0.013)**	**(−0.034,0.094)**	**(−0.174, −0.050)**	**(−0.089,0.027)**
**BMI change**	**0.041**	**0.009**	**−0.054**	**0.068**	**−0.014**	**0.016**	**0.004**	**−0.023**
**5–5.5y**	**(−0.013,0.096)**	**(−0.047,0.064)**	**(−0.125,0.017)**	**(−0.002,0.135)**	**(−0.087,0.059)**	**(−0.043,0.076)**	**(−0.075,0.082)**	**(−0.109,0.063)**
**BMI change**	**−0.017**	**0.008**	**−0.006**	**−0.026**	**−0.017**	**−0.016**	**0.017**	**−0.009**
**5.5–6.5y**	**(−0.072,0.038)**	**(−0.050,0.066)**	**(−0.069,0.058)**	**(−0.101,0.049)**	**(−0.087,0.054)**	**(−0.083,0.051)**	**(−0.049,0.084)**	**(−0.079,0.061)**
BMI change	−0.011	−0.070	−0.023	−0.099	0.034	0.011	0.056	0.039
6.5–7y	(−0.114,0.093)	(−0.174,0.035)	(−0.144,0.097)	(−0.231,0.034)	(−0.100,0.168)	(−0.106,0.128)	(−0.065,0.176)	(−0.074,0.151)
BMI change	0.043	0.002	0.013	0.016	0.021	−0.011	0.063	0.045
7–8.5y	(−0.016,0.102)	(−0.058,0.063)	(−0.063,0.090)	(−0.066,0.098)	(−0.049,0.090)	(−0.077,0.054)	(−0.012,0.128)	(−0.050,0.139)
BMI change	−0.008	0.043	−0.064	−0.004	−0.039	−0.028	0.086	0.011
8.5–10y	(−0.113,0.098)	(−0.061,0.148)	(−0.178,0.050)	(−0.114,0.106)	(−0.163,0.085)	(−0.177,0.122)	(−0.015,0.187)	(−0.099,0.121)

All variables were standardised prior to analysis. Coefficients represent the standard deviation change in the cardiovascular risk factor associated with a one standard deviation increase in the rate of PI/BMI change.

**Bold text** indicates that PI/BMI is, on average, declining during that period.

Adjusted for age, previous periods of PI/BMI change, gender, maternal and partner education, household social class, maternal age, height, gestational age at birth, maternal and partner BMI, maternal and partner smoking in pregnancy, age at clinic attendance, pubertal stage at measurement of outcome, DXA-assessed fat mass at age 15, height and height squared at the time of DXA scan.

PI/BMI change periods:

BMI change 2–5y: 24 and 60 months for boys, 24 and 56 months for girls.

BMI change 5–5.5y: 60 and 65 months for boys, 56 and 67 months for girls.

BMI change 5.5–6.5y: 65 and 75 months for boys, 67 and 73 months for girls.

BMI change 6.5–7y: 75 and 81 months for boys, 73 and 79 months for girls.

BMI change 7–8.5y: 81 and 103 months for boys, 79 and 105 months for girls.

BMI change 8.5–10y: 103 and 120 months for boys, 105 and 120 months for girls.

There was some suggestion of associations between PI/BMI changes and later cardiovascular risk factors being stronger in boys than in girls. Associations were considerably weaker for DBP compared with SBP. The magnitude of associations was stronger for HDLc and log triglycerides than for LDLc. Associations between adiposity trajectories and glucose were weak.

## Discussion

### Main findings

All periods of PI/BMI change between birth and age 10 were associated with fat mass at age 15. The period between 0–10 years with the strongest association between PI/BMI change and DXA-assessed fat mass in adolescence was age 2–5 years for girls, and 5–5.5 years for boys. Findings from the Earlybird study indicated that most increases in weight gain z-scores before the age of 9 occurred prior to age 5.[11] As a result of this study, there was suggestion that obesity prevention efforts should be focused primarily on pre-school children. Whilst we demonstrate that changes in BMI between 2–5 years do appear to be strongly associated with later adiposity, our findings also suggest that BMI changes in later childhood were also strongly associated with increased fat mass, as well as with a range of cardiovascular risk factors at age 15. This would argue for obesity prevention efforts aimed at children of all ages.

There was little evidence of associations between PI changes between 0–2 years or BMI changes in early childhood and cardiovascular risk factors at age 15 after adjustment for potential confounding factors. BMI changes later in childhood (7–8.5 and 8.5–10 years) did demonstrate associations with cardiovascular risk factors. These associations were largely attenuated by adjustment for fat mass at age 15, i.e. associations between early BMI changes and later cardiovascular risk factors are largely mediated by the associations of childhood growth with fat mass in adolescence.[10] This may imply that tracking of adiposity across infancy and childhood to adolescence is the main explanation for associations between early growth and later cardiovascular risk factors.

Our analyses showed that PI/BMI changes in childhood are only weakly associated with DBP and fasting glucose at age 15. This is consistent with a recent cross-sectional study of 6–17 year olds in the US Nutritional Examination Survey, which demonstrated associations across most of the BMI distribution with fasting lipids, but the odds of elevated fasting glucose only increased once BMI was above the 99^th^ age and gender specific percentile, and odds for elevated DBP only increased after the 95^th^ percentile.[20]

To our knowledge, no other studies have demonstrated stronger associations between adiposity changes in childhood and later cardiovascular risk factors in males compared with females; further studies are needed to validate this result.

Our study has explored the effects of PI/BMI changes across childhood on fat mass and a wide range of cardiovascular risk factors in adolescence using a large contemporary cohort study with repeat measures of PI/BMI across childhood used to model individual adiposity trajectories. Our modelling approach has allowed us to explore whether there are periods of PI/BMI change in childhood that are particularly strongly associated with later fat mass and cardiovascular risk factors. To our knowledge this is the first study to explore changes in adiposity from birth to late childhood and their association with adolescent cardiovascular risk factors. One limitation of modelling adiposity trajectories using BMI is that BMI is an imperfect measure of adiposity in childhood, in particular in infancy; it is associated with both lean and fat mass in young children. However, we have attempted to minimise this limitation by modelling PI from birth to two years, which reduces the correlation with height. Furthermore, evidence from this cohort suggests that BMI has similar magnitudes of association to cardiovascular risk factors in childhood as does total fat mass assessed by DXA or waist circumference[21], suggesting that BMI may adequately assess adiposity in childhood. As in all cohort studies, there has been loss to follow up of ALSPAC participants. However, we do not believe that PI/BMI changes would be differently associated with cardiovascular risk factors in those who were lost to follow-up. Our multivariable multiple imputation analysis results were similar to complete case analyses, suggesting that there are no major problems with selection bias between those with no missing data (including on fasting bloods) and the whole eligible cohort who attended the follow-up clinic.

In summary, our results indicate that PI/BMI changes at all ages from birth to age 10 are associated with fat mass at age 15, and BMI changes in later childhood are associated with a range of cardiovascular risk factors at age 15. Associations between PI/BMI changes between birth and ten years and cardiovascular risk factors at age 15 were largely mediated by fat mass at age 15. This mediation by adiposity at age 15 could potentially reflect tracking of adiposity through infancy and childhood as the main reason why greater adiposity in early life is related to cardiovascular risk. Collectively, our results would argue for obesity prevention efforts incorporating children of all ages rather than focusing on specific ages.

## Supporting Information

Figure S1 Boys Associations between adiposity trajectories and SBP**.** Graphs of coefficients and 95% confidence intervals from standardised linear regressions of cardiovascular risk factors at age 15 on PI/BMI trajectories (age in years along x-axis is the mid-point of PI/BMI change periods; coefficients are adjusted for confounders and previous PI/BMI changes, i.e. Model 3 in Tables)
(TIF)

Figure S2 Boys Associations between adiposity trajectories and DBP**.** Graphs of coefficients and 95% confidence intervals from standardised linear regressions of cardiovascular risk factors at age 15 on PI/BMI trajectories (age in years along x-axis is the mid-point of PI/BMI change periods; coefficients are adjusted for confounders and previous PI/BMI changes, i.e. Model 3 in Tables)
(TIF)

Figure S3 Boys Associations between adiposity trajectories and LDLc**.** Graphs of coefficients and 95% confidence intervals from standardised linear regressions of cardiovascular risk factors at age 15 on PI/BMI trajectories (age in years along x-axis is the mid-point of PI/BMI change periods; coefficients are adjusted for confounders and previous PI/BMI changes, i.e. Model 3 in Tables)
(TIF)

Figure S4 Boys Associations between adiposity trajectories and HDLc**.** Graphs of coefficients and 95% confidence intervals from standardised linear regressions of cardiovascular risk factors at age 15 on PI/BMI trajectories (age in years along x-axis is the mid-point of PI/BMI change periods; coefficients are adjusted for confounders and previous PI/BMI changes, i.e. Model 3 in Tables)
(TIF)

Figure S5 Boys Associations between adiposity trajectories and Ln CRP**.** Graphs of coefficients and 95% confidence intervals from standardised linear regressions of cardiovascular risk factors at age 15 on PI/BMI trajectories (age in years along x-axis is the mid-point of PI/BMI change periods; coefficients are adjusted for confounders and previous PI/BMI changes, i.e. Model 3 in Tables)
(TIF)

Figure S6 Boys Associations between adiposity trajectories and Ln Insulin**.** Graphs of coefficients and 95% confidence intervals from standardised linear regressions of cardiovascular risk factors at age 15 on PI/BMI trajectories (age in years along x-axis is the mid-point of PI/BMI change periods; coefficients are adjusted for confounders and previous PI/BMI changes, i.e. Model 3 in Tables)
(TIF)

Figure S7 Boys Associations between adiposity trajectories and Glucose**.** Graphs of coefficients and 95% confidence intervals from standardised linear regressions of cardiovascular risk factors at age 15 on PI/BMI trajectories (age in years along x-axis is the mid-point of PI/BMI change periods; coefficients are adjusted for confounders and previous PI/BMI changes, i.e. Model 3 in Tables)
(TIF)

Figure S8 Girls Associations between adiposity trajectories and SBP**.** Graphs of coefficients and 95% confidence intervals from standardised linear regressions of cardiovascular risk factors at age 15 on PI/BMI trajectories (age in years along x-axis is the mid-point of PI/BMI change periods; coefficients are adjusted for confounders and previous PI/BMI changes, i.e. Model 3 in Tables)
(TIF)

Figure S9 Girls Associations between adiposity trajectories and DBP**.** Graphs of coefficients and 95% confidence intervals from standardised linear regressions of cardiovascular risk factors at age 15 on PI/BMI trajectories (age in years along x-axis is the mid-point of PI/BMI change periods; coefficients are adjusted for confounders and previous PI/BMI changes, i.e. Model 3 in Tables)
(TIF)

Figure S10 Girls Associations between adiposity trajectories and LDLc**.** Graphs of coefficients and 95% confidence intervals from standardised linear regressions of cardiovascular risk factors at age 15 on PI/BMI trajectories (age in years along x-axis is the mid-point of PI/BMI change periods; coefficients are adjusted for confounders and previous PI/BMI changes, i.e. Model 3 in Tables)
(TIF)

Figure S11 Girls Associations between adiposity trajectories and HDLc**.** Graphs of coefficients and 95% confidence intervals from standardised linear regressions of cardiovascular risk factors at age 15 on PI/BMI trajectories (age in years along x-axis is the mid-point of PI/BMI change periods; coefficients are adjusted for confounders and previous PI/BMI changes, i.e. Model 3 in Tables)
(TIF)

Figure S12 Girls Associations between adiposity trajectories and Ln CRP**.** Graphs of coefficients and 95% confidence intervals from standardised linear regressions of cardiovascular risk factors at age 15 on PI/BMI trajectories (age in years along x-axis is the mid-point of PI/BMI change periods; coefficients are adjusted for confounders and previous PI/BMI changes, i.e. Model 3 in Tables)
(TIF)

Figure S13 Girls Associations between adiposity trajectories and Ln Insulin**.** Graphs of coefficients and 95% confidence intervals from standardised linear regressions of cardiovascular risk factors at age 15 on PI/BMI trajectories (age in years along x-axis is the mid-point of PI/BMI change periods; coefficients are adjusted for confounders and previous PI/BMI changes, i.e. Model 3 in Tables)
(TIF)

Figure S14 Girls Associations between adiposity trajectories and Glucose**.** Graphs of coefficients and 95% confidence intervals from standardised linear regressions of cardiovascular risk factors at age 15 on PI/BMI trajectories (age in years along x-axis is the mid-point of PI/BMI change periods; coefficients are adjusted for confounders and previous PI/BMI changes, i.e. Model 3 in Tables)
(TIF)

File S1 Details of measurement of height and weight at research clinics
(DOCX)

File S2 Details of measurement of cardiovascular risk factors at age 15 clinic
(DOCX)

File S3 Measurement of confounding factors
(DOCX)

File S4 Details of statistical modelling of PI/BMI trajectories
(DOCX)

File S5 Details of multiple imputation procedure
(DOCX)

Table S1 Description of singleton ALSPAC participants included in our analyses, and comparisons with the original cohort
(DOCX)

Table S2 Actual ponderal index (PI) and body mass index (BMI) measurements, difference between actual measurements and those predicted by the multilevel models, and estimated rates of adiposity change in all ALSPAC participants with at least 1 adiposity measure
(DOCX)

Table S3 Adiposity trajectories from birth to ten years and their association with DXA-assessed total body fat mass at age 15 years, with multiple imputation
(DOCX)

Table S4 Adiposity trajectories from birth to ten years and their association with systolic blood pressure at age 15 years, with multiple imputation
(DOCX)

Table S5 Adiposity trajectories from birth to ten years and their association with diastolic blood pressure at age 15 years, with multiple imputation
(DOCX)

Table S6 Adiposity trajectories from birth to ten years and their association with LDLc at age 15 years, with multiple imputation
(DOCX)

Table S7 Adiposity trajectories from birth to ten years and their association with HDLc at age 15 years, with multiple imputation
(DOCX)

Table S8 Adiposity trajectories from birth to ten years and their association with Ln triglycerides at age 15 years, with multiple imputation
(DOCX)

Table S9 Adiposity trajectories from birth to ten years and their association with Ln CRP at age 15 years, with multiple imputation
(DOCX)

Table S10 Adiposity trajectories from birth to ten years and their association with Ln insulin at age 15 years, with multiple imputation
(DOCX)

Table S11 Adiposity trajectories from birth to ten years and their association with glucose at age 15 years, with multiple imputation
(DOCX)

Table S12 Associations between cardiovascular risk factors and DXA-assessed fat mass, both measured at age 15 years
(DOCX)

## References

[1] Wang Y, Lobstein T (2006). Worldwide trends in childhood overweight and obesity.. International Journal of Pediatric Obesity.

[2] Ogden CL, Carroll MD, Flegal KM (2008). High body mass index for age among US children and adolescents.. Journal of the American Medical Association.

[3] Kipping RR, Jago R, Lawlor DA (2008). Obesity in children. Part 1: Epidemiology, measurement, risk factors, and screening.. British Medical Journal.

[4] Sundblom E, Petzold M, Rasmussen F, Callmer E, Lissner L (2008). Childhood overweight and obesity prevalences levelling off in Stockholm but socioeconomic differences persist.. International Journal of Obesity.

[5] Owen CG, Whincup PH, Orfei L, Chou Q-A, Rudnicka AR (2009). Is body mass index before middle age related to coronary heart disease risk in later life? Evidence from observational studies.. International Journal of Obesity.

[6] Must A, Strauss RS (1999). Risks and consequences of childhood and adolescent obesity.. International Journal of Obesity.

[7] Sinha R, Fisch G, Teague B, Tamborlane WV, Banyas B (2002). Prevalence of Impaired Glucose Tolerance among Children and Adolescents with Marked Obesity.. N Engl J Med.

[8] Fraser A, Longnecker MP, Lawlor DA (2007). Prevalence of Elevated Alanine Aminotransferase Among US Adolescents and Associated Factors: NHANES 1999-2004.. Gastroenterology.

[9] Freedman DS, Dietz WH, Srinivasan SR, Berenson GS (1999). The relation of overweight to cardiovascular risk factors among children and adolescents: the Bogalusa Heart Study.. Pediatrics.

[10] Singh AS, Mulder C, Twisk JW, Van Mechelen W, Chinapaw MJM (2008). Tracking of childhood overweight into adulthood: a systematic review of the literature.. Obesity Reviews.

[11] Gardner DSL, Hosking J, Metcalf BS, Jeffery AN, Voss LD (2009). Contribution of Early Weight Gain to Childhood Overweight and Metabolic Health: A Longitudinal Study (EarlyBird 36).. Pediatrics.

[12] Ong KK, Ahmed ML, Emmett PM, Preece MA, Dunger DB (2000). Association between postnatal catch-up growth and obesity in childhood: prospective cohort study.. British Medical Journal.

[13] Ong KK, Loos RJF (2006). Rapid infancy weight gain and subsequent obesity: Systematic reviews and hopeful suggestions.. Acta Paediatrica.

[14] Ong KK, Emmett PM, Northstone K, Golding J, Rogers I (2009). Infancy weight gain predicts childhood body fat and age at menarche.. J Clin Endocrinol Metab.

[15] Golding J, Pembrey M, Jones R, the ALSPAC Study Team. (2001). ALSPAC - The Avon Longitudinal Study of Parents and Children I. Study Methodology.. Paediatric & Perinatal Epidemiology.

[16] Howe LD, Tilling K, Lawlor DA (2009). Accuracy of height and weight data from child health records.. Archives of Disease in Childhood.

[17] Dubois L, Girad M (2007). Accuracy of maternal reports of pre-schoolers' weights and heights as estimates of BMI values.. Int J Epidemiol.

[18] StataCorp (2010). Stata 11.0, version Texas: StataCorp.

[19] Cole TJ (2004). Children grow and horses race: is the adiposity rebound a critical period for later obesity?. BMC Pediatrics.

[20] Skinner AC, Mayer ML, Flower K, Perrin EM, Weinberger M (2009). Using BMI to Determine Cardiovascular Risk in Childhood: How Do the BMI Cutoffs Fare?. Pediatrics.

[21] Falaschetti E, Hingorani AD, Jones A, Charakida M, Finer N (2010). Adiposity and cardiovascular risk factors in a large contemporary population of pre-pubertal children.. European Heart Journal (In Press).

