# Supplementary figures and images for: Changes in Ponderal Index and Body Mass Index across Childhood and Their Associations with Fat Mass and Cardiovascular Risk Factors at Age 15
Source: PLoS One. 2010 Dec 8;5(12):e15186. doi: 10.1371/journal.pone.0015186 (PMC2999567; doi:10.1371/journal.pone.0015186)

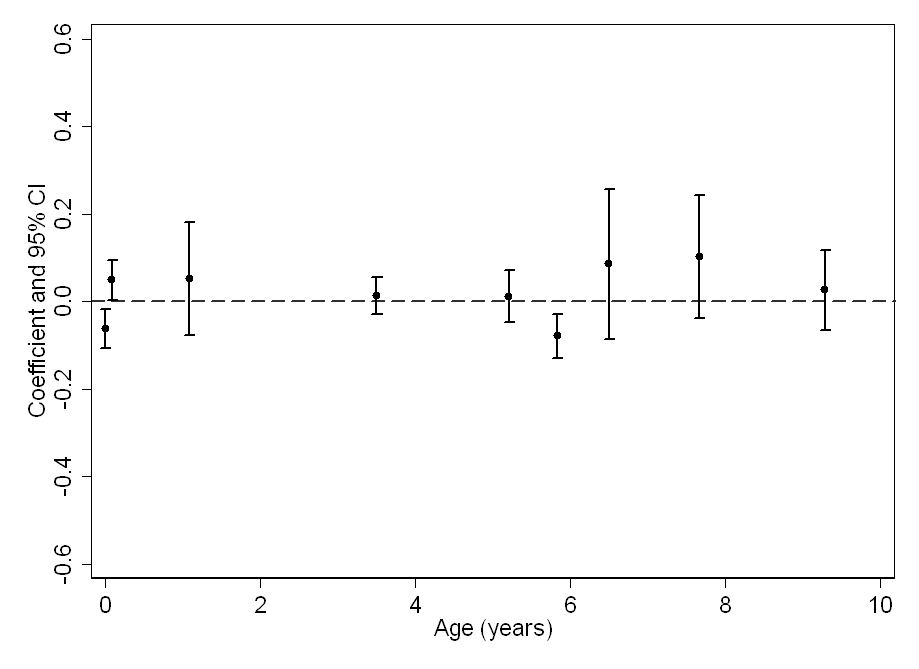

Supplement: Figure S1 — Boys Associations between adiposity trajectories and SBP. Graphs of coefficients and 95% confidence intervals from standardised linear regressions of cardiovascular risk factors at age 15 on PI/BMI trajectories (age in years along x-axis is the mid-point of PI/BMI change periods; coefficients are adjusted for confounders and previous PI/BMI changes, i.e. Model 3 in Tables) (TIF) [file pone.0015186.s001.tif]

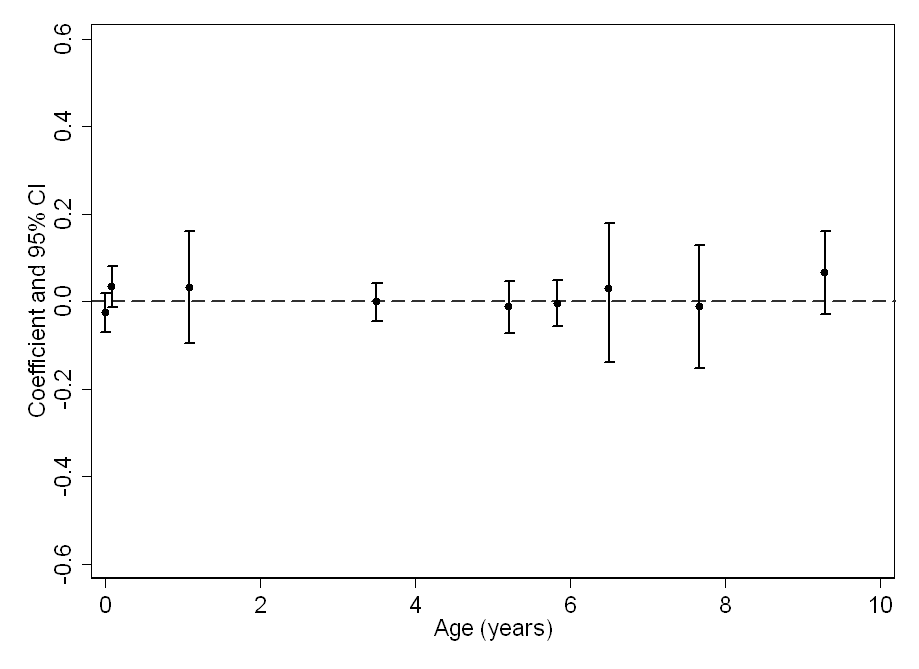

Supplement: Figure S2 — Boys Associations between adiposity trajectories and DBP. Graphs of coefficients and 95% confidence intervals from standardised linear regressions of cardiovascular risk factors at age 15 on PI/BMI trajectories (age in years along x-axis is the mid-point of PI/BMI change periods; coefficients are adjusted for confounders and previous PI/BMI changes, i.e. Model 3 in Tables) (TIF) [file pone.0015186.s002.tif]

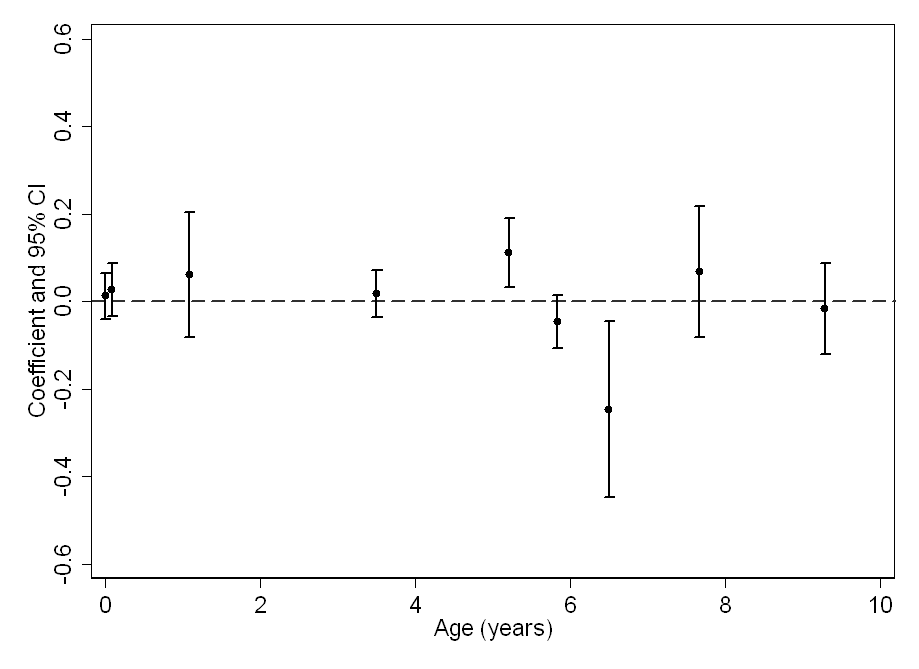

Supplement: Figure S3 — Boys Associations between adiposity trajectories and LDLc. Graphs of coefficients and 95% confidence intervals from standardised linear regressions of cardiovascular risk factors at age 15 on PI/BMI trajectories (age in years along x-axis is the mid-point of PI/BMI change periods; coefficients are adjusted for confounders and previous PI/BMI changes, i.e. Model 3 in Tables) (TIF) [file pone.0015186.s003.tif]

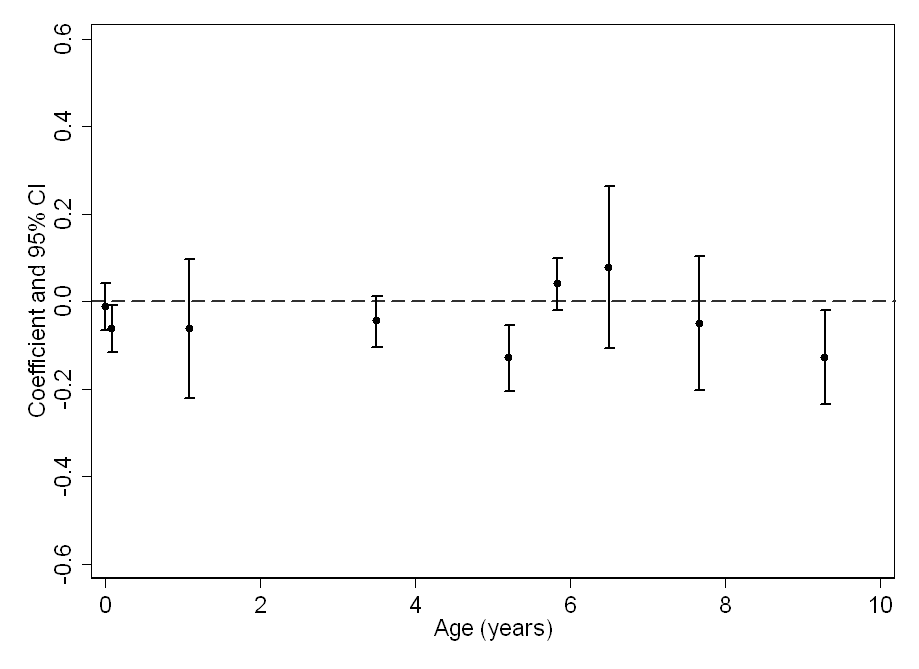

Supplement: Figure S4 — Boys Associations between adiposity trajectories and HDLc. Graphs of coefficients and 95% confidence intervals from standardised linear regressions of cardiovascular risk factors at age 15 on PI/BMI trajectories (age in years along x-axis is the mid-point of PI/BMI change periods; coefficients are adjusted for confounders and previous PI/BMI changes, i.e. Model 3 in Tables) (TIF) [file pone.0015186.s004.tif]

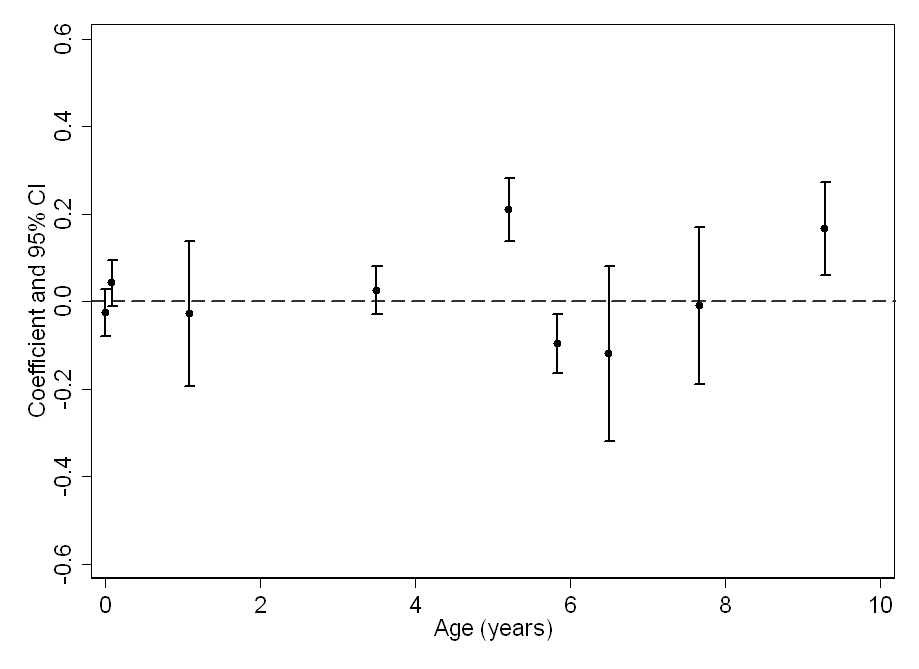

Supplement: Figure S5 — Boys Associations between adiposity trajectories and Ln CRP. Graphs of coefficients and 95% confidence intervals from standardised linear regressions of cardiovascular risk factors at age 15 on PI/BMI trajectories (age in years along x-axis is the mid-point of PI/BMI change periods; coefficients are adjusted for confounders and previous PI/BMI changes, i.e. Model 3 in Tables) (TIF) [file pone.0015186.s005.tif]

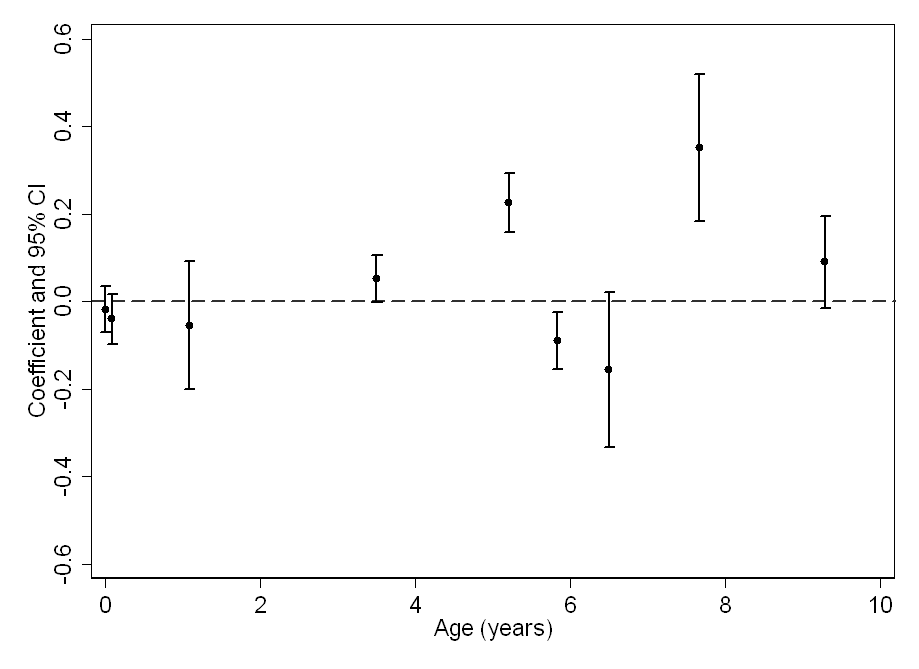

Supplement: Figure S6 — Boys Associations between adiposity trajectories and Ln Insulin. Graphs of coefficients and 95% confidence intervals from standardised linear regressions of cardiovascular risk factors at age 15 on PI/BMI trajectories (age in years along x-axis is the mid-point of PI/BMI change periods; coefficients are adjusted for confounders and previous PI/BMI changes, i.e. Model 3 in Tables) (TIF) [file pone.0015186.s006.tif]

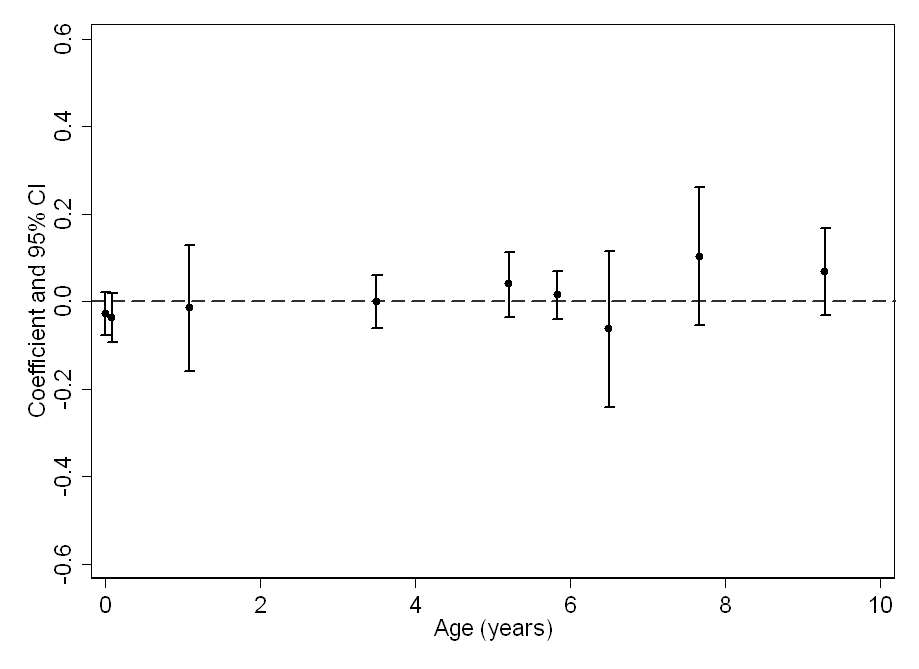

Supplement: Figure S7 — Boys Associations between adiposity trajectories and Glucose. Graphs of coefficients and 95% confidence intervals from standardised linear regressions of cardiovascular risk factors at age 15 on PI/BMI trajectories (age in years along x-axis is the mid-point of PI/BMI change periods; coefficients are adjusted for confounders and previous PI/BMI changes, i.e. Model 3 in Tables) (TIF) [file pone.0015186.s007.tif]

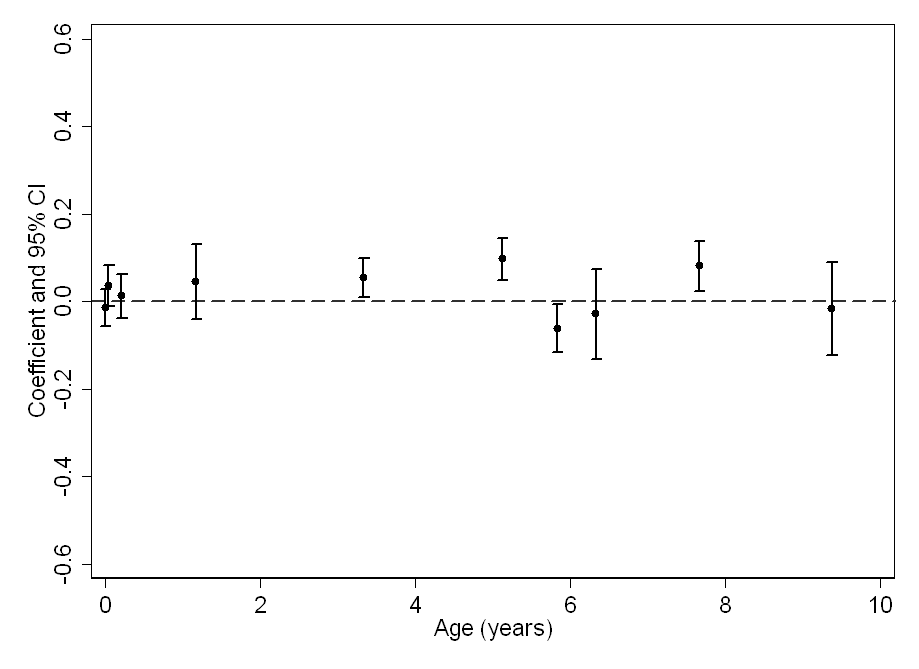

Supplement: Figure S8 — Girls Associations between adiposity trajectories and SBP. Graphs of coefficients and 95% confidence intervals from standardised linear regressions of cardiovascular risk factors at age 15 on PI/BMI trajectories (age in years along x-axis is the mid-point of PI/BMI change periods; coefficients are adjusted for confounders and previous PI/BMI changes, i.e. Model 3 in Tables) (TIF) [file pone.0015186.s008.tif]

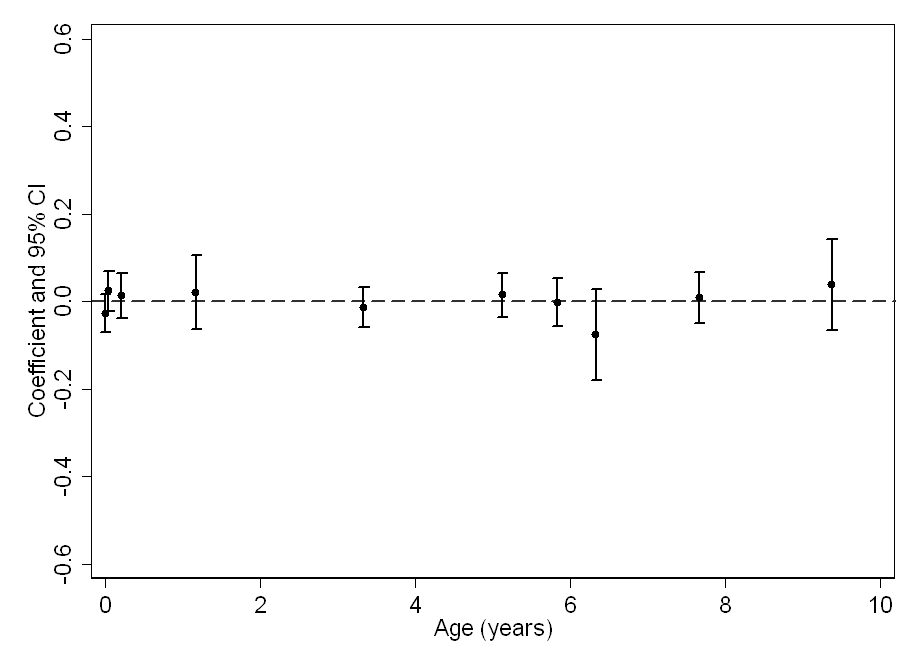

Supplement: Figure S9 — Girls Associations between adiposity trajectories and DBP. Graphs of coefficients and 95% confidence intervals from standardised linear regressions of cardiovascular risk factors at age 15 on PI/BMI trajectories (age in years along x-axis is the mid-point of PI/BMI change periods; coefficients are adjusted for confounders and previous PI/BMI changes, i.e. Model 3 in Tables) (TIF) [file pone.0015186.s009.tif]

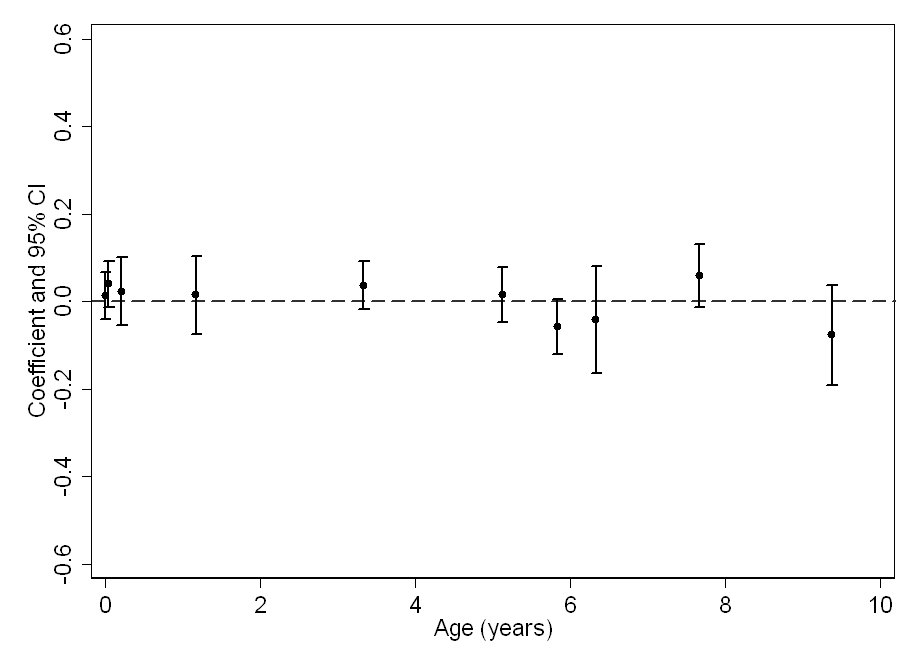

Supplement: Figure S10 — Girls Associations between adiposity trajectories and LDLc. Graphs of coefficients and 95% confidence intervals from standardised linear regressions of cardiovascular risk factors at age 15 on PI/BMI trajectories (age in years along x-axis is the mid-point of PI/BMI change periods; coefficients are adjusted for confounders and previous PI/BMI changes, i.e. Model 3 in Tables) (TIF) [file pone.0015186.s010.tif]

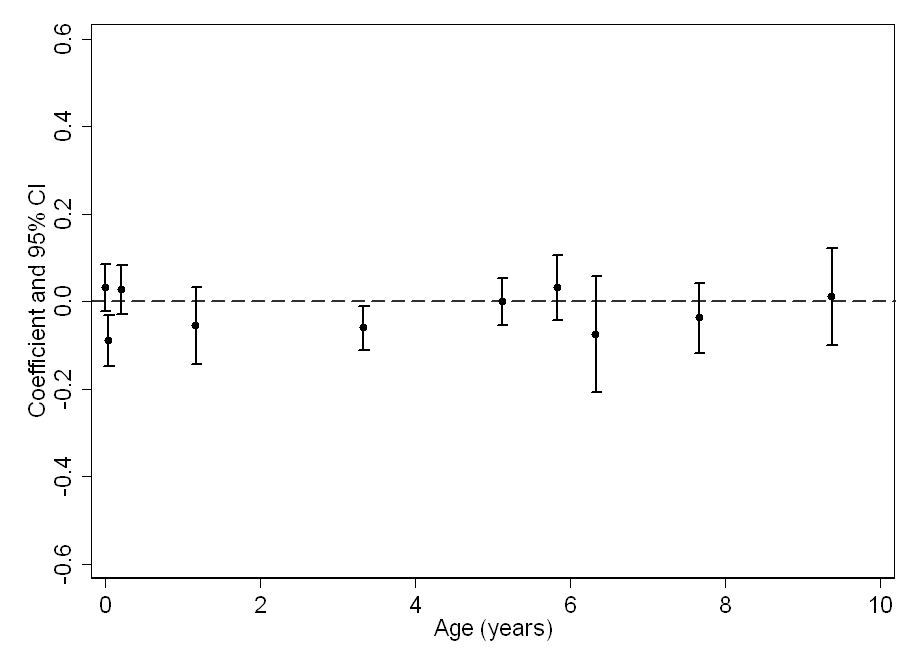

Supplement: Figure S11 — Girls Associations between adiposity trajectories and HDLc. Graphs of coefficients and 95% confidence intervals from standardised linear regressions of cardiovascular risk factors at age 15 on PI/BMI trajectories (age in years along x-axis is the mid-point of PI/BMI change periods; coefficients are adjusted for confounders and previous PI/BMI changes, i.e. Model 3 in Tables) (TIF) [file pone.0015186.s011.tif]

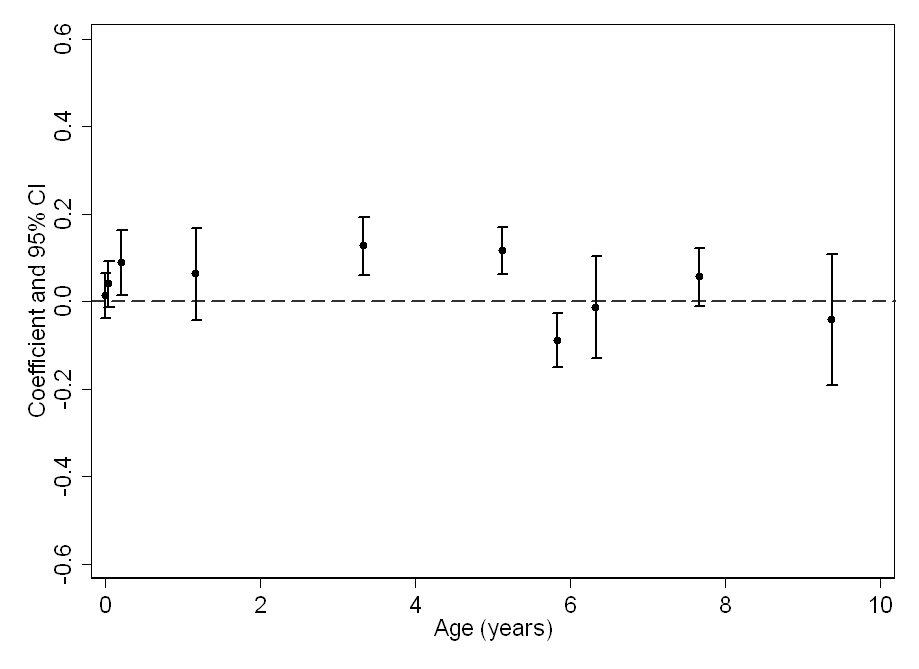

Supplement: Figure S12 — Girls Associations between adiposity trajectories and Ln CRP. Graphs of coefficients and 95% confidence intervals from standardised linear regressions of cardiovascular risk factors at age 15 on PI/BMI trajectories (age in years along x-axis is the mid-point of PI/BMI change periods; coefficients are adjusted for confounders and previous PI/BMI changes, i.e. Model 3 in Tables) (TIF) [file pone.0015186.s012.tif]

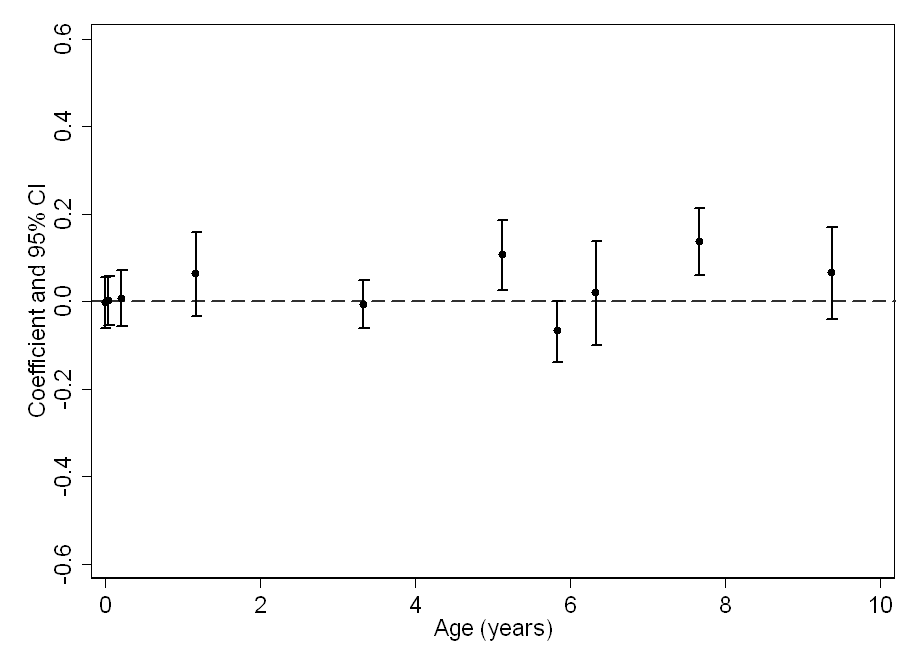

Supplement: Figure S13 — Girls Associations between adiposity trajectories and Ln Insulin. Graphs of coefficients and 95% confidence intervals from standardised linear regressions of cardiovascular risk factors at age 15 on PI/BMI trajectories (age in years along x-axis is the mid-point of PI/BMI change periods; coefficients are adjusted for confounders and previous PI/BMI changes, i.e. Model 3 in Tables) (TIF) [file pone.0015186.s013.tif]

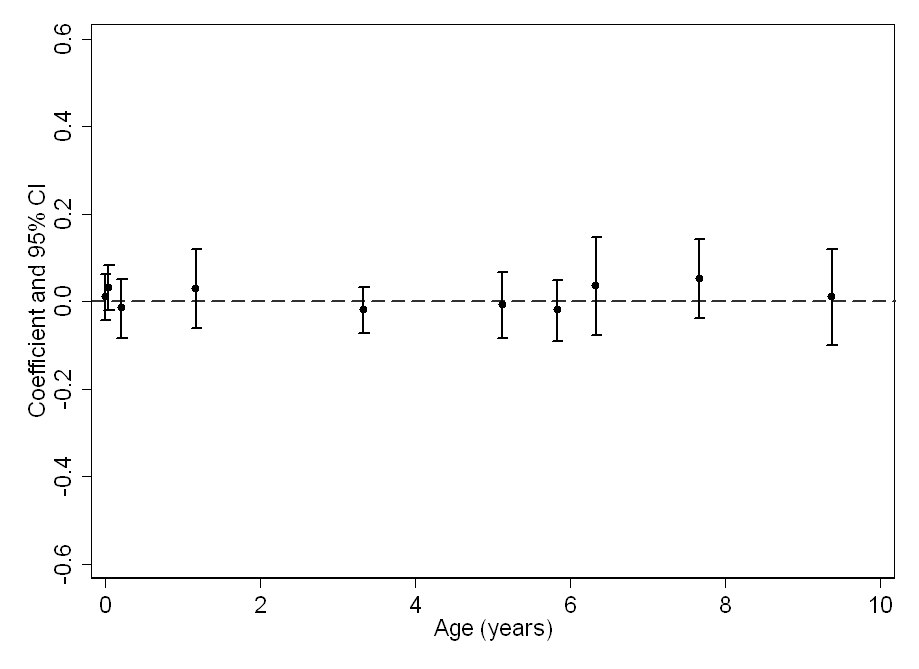

Supplement: Figure S14 — Girls Associations between adiposity trajectories and Glucose. Graphs of coefficients and 95% confidence intervals from standardised linear regressions of cardiovascular risk factors at age 15 on PI/BMI trajectories (age in years along x-axis is the mid-point of PI/BMI change periods; coefficients are adjusted for confounders and previous PI/BMI changes, i.e. Model 3 in Tables) (TIF) [file pone.0015186.s014.tif]
