# Supplementary material for: Changes in Ponderal Index and Body Mass Index across Childhood and Their Associations with Fat Mass and Cardiovascular Risk Factors at Age 15
Source: PLoS One. 2010 Dec 8;5(12):e15186. doi: 10.1371/journal.pone.0015186 (PMC2999567; doi:10.1371/journal.pone.0015186)
Supplement: File S1 — Details of measurement of height and weight at research clinics (DOCX) [file pone.0015186.s015.docx]

**Supporting File 1: Details of measurement of height and weight at research clinics**

At the clinics between four months and five years, crown-heel length for children aged 4 to 25 months was measured using a Harpenden Neonatometer and from 25 months onwards standing height was measured using a Leicester Height Measure; weight was measured using Fereday 100kg combined scale (4 month clinic), Soenhle scale or Seca scale model 724 (8 month clinic), Seca 724 or Seca 835 (12 month clinic), Seca 835 (18 months onwards). From age seven years upwards, all children were invited to annual clinics, at which standing height was measured to the last complete mm using the Harpenden Stadiometer and weight was measured to the nearest 0.1kg using the Tanita Body Fat Analyser (Model TBF 305).
