# Supplementary material for: Changes in Ponderal Index and Body Mass Index across Childhood and Their Associations with Fat Mass and Cardiovascular Risk Factors at Age 15
Source: PLoS One. 2010 Dec 8;5(12):e15186. doi: 10.1371/journal.pone.0015186 (PMC2999567; doi:10.1371/journal.pone.0015186)
Supplement: File S2 — Details of measurement of cardiovascular risk factors at age 15 clinic (DOCX) [file pone.0015186.s016.docx]

**Supporting File 2: Details of measurement of cardiovascular risk factors at age 15 clinic**

Participants were asked to fast overnight (for those attending in the morning) or for at least 6 hours for those attending after lunch. Blood samples were immediately spun and frozen at –80^o^C. Measurements were assayed in batches shortly (3-9 months) after samples were taken with no previous freeze-thaw cycles during this period. Plasma lipids (total cholesterol, triglycerides and HDL-C) were performed by modification of the standard Lipid Research Clinics Protocol using enzymatic reagents for lipid determination. LDL-C was calculated using the Friedwald equation (LDLc = total cholesterol minus (HDLc + triglycerides×0.45)). Insulin was measured by an ELISA (Mercodia, Uppsala, Sweden) automated microparticle enzyme immunoassay that does not cross-react with proinsulin and plasma glucose was measured by automated enzymatic (Hexokinase). Blood pressure was measured using a Dinamap 9301 Vital Signs Monitor (Morton Medical, London, UK). Arm circumference was measured prior to blood pressure assessment and used to determine the correct cuff size. Two readings of systolic and diastolic BP (SBP and DBP) were recorded, with the child at rest and their arm supported, and the mean of each was used.
