# Supplementary material for: Changes in Ponderal Index and Body Mass Index across Childhood and Their Associations with Fat Mass and Cardiovascular Risk Factors at Age 15
Source: PLoS One. 2010 Dec 8;5(12):e15186. doi: 10.1371/journal.pone.0015186 (PMC2999567; doi:10.1371/journal.pone.0015186)
Supplement: File S3 — Measurement of confounding factors (DOCX) [file pone.0015186.s017.docx]

**Supporting File 3: Measurement of confounding factors**

A questionnaire at 32 weeks gestation asked mothers to report their and their partner’s educational attainment, which was categorised as below O-Level (Ordinary Level; exams taken in different subjects usually at age 15-16 at the completion of legally required school attendance, equivalent to today’s UK General Certificate of Secondary Education), O-Level only, A-Level (Advanced-Level; exams taken in different subjects usually at age 18), or university degree or above. Household social class is measured as the highest of the mother’s or her partner’s occupational social class using data on job title and details of occupation collected about the mother and her partner from the mother’s questionnaire at 32 weeks gestation. Social class is derived using the standard occupational classification (SOC) codes developed by the United Kingdom Office of Population Census and Surveys. Social class is categorised as I (professional), II (managerial and technical), III non-manual (skilled occupations, non-manual), III manual (skilled occupations, manual), IV (part skilled occupations) and V (unskilled occupations). In our analyses, armed forces were excluded since this represents a mixture of officers and lower rank staff, and the social class variable was collapsed into a binary indicator of manual (classes III M, IV and V) or non-manual (classes I, II, III NM). Maternal age was reported in the antenatal mother’s questionnaires. Gestational age at birth was estimated from clinical records. For all live births the gestation was recorded in a variety of ways on the “stork database”, using last menstrual period, paediatric assessment, obstetric assessment and ultrasound assessment. Maternal BMI was calculated using self-reported height and pre-pregnancy weight from a questionnaire administered at 12 weeks gestation, partners’ also reported their own heights and weights in questionnaires at 12 weeks gestation. Maternal and partner self-reported smoking in pregnancy were coded as ‘any’ or ‘no’ smoking at any time during pregnancy, using measures from several pregnancy questionnaires. Age of child at measurement of outcome was assessed in complete month upon attendance at the clinic. Pubertal stage was assessed using questionnaires containing line drawings and questions based on stages described by Tanner[1,2], which were completed by the child’s parent or guardian (most often mother) within one year of attendance at the clinic; Tanner stage ranges between I and V, with I being least sexually mature. Height was predicted from multilevel models similar to those used to model PI and BMI; individual growth trajectories were modelled using linear spline random effects models with knot points at 3 months, 10 months, and 29 months for girls,2 months, 11 months, and 32 months for boys. Individual coefficients from these models were used to predict height at the exact ages of each adiposity trajectory knot point. Regressions on the adiposity trajectory variables were then adjusted for the predicted height at the corresponding age of the oldest adiposity trajectory period included in each model.
