# Supplementary material for: Changes in Ponderal Index and Body Mass Index across Childhood and Their Associations with Fat Mass and Cardiovascular Risk Factors at Age 15
Source: PLoS One. 2010 Dec 8;5(12):e15186. doi: 10.1371/journal.pone.0015186 (PMC2999567; doi:10.1371/journal.pone.0015186)
Supplement: File S4 — Details of statistical modelling of PI/BMI trajectories (DOCX) [file pone.0015186.s018.docx]

**Supporting File 4: Details of statistical modelling of PI/BMI trajectories**

The standard way of modelling the relationship between a continuous outcome (in our example ponderal index (PI) and body mass index (BMI)) and a continuous exposure (in our example age) would be to fit a polynomial curve (i.e. age raised to the appropriate power). The patterns of changes in adiposity across childhood, however, follow a complex pattern and may not be accurately represented by a simple polynomial curve. For this reason, we used fractional polynomials to find the best-fitting average PI and BMI trajectory for each of our models (PI from birth to two years, BMI from two to ten years, separately for boys and girls). Fractional polynomials is an approach to modelling the relationship between an outcome and one or more continuous covariates in which the continuous covariate is raised to a large number of combinations of powers, resulting in a wide range of possible curves and offering more flexibility than standard polynomial approaches [3]. In our example, age was raised to various combinations of powers, from which we selected the best fitting curve (the one with the lowest likelihood value). Although fractional polynomials provide a flexible way to examine such relationships, they do not provide parameters that are clinically relevant, easily interpreted, or readily used to assess the impact of different growth periods on later outcomes. We therefore used the best-fitting fractional polynomial to derive a piecewise linear spline model. From the best fitting curves, we derived approximate knot-points for the linear spline model, i.e. the approximate turning points of the curve between which changes in height were approximately linear.

To optimise the knot points, we fitted a series of models with the knot points placed at 1 month intervals around the estimated knot point identified from the shape of the fractional polynomial curve. The model with the lowest likelihood value was selected as the knot point for the final model. This confirmed that there were:

This modelling confirmed that there were:

- two periods of PI change in boys (birth to 2 months and 2 to 24 months)
- three periods of PI change in girls (birth to 1 month, 1 to 4 months, and 4 to 24 months)
- six periods of BMI change in both girls and boys (in boys: 24 to 56, 56 to 67, 67 to 73, 73 to 79, 79 to 105, and 105 to 120 months; in girls: 24 to 60, 60 to 65, 65 to 75, 75 to 81, 81 to 103, and 103 to 120 months).

Individual-level random effects allowed intercepts and slopes for each period to vary between individuals.

Actual and predicted measurements (from the multilevel models) were compared to assess model fit. We also checked for auto-correlation (residual correlation between an individual’s measurements as a decreasing function of the difference in the age at measurement, a phenomenon that can cause problems in growth models, particularly when repeated measurements are close together in time as they are in these analyses [4]). We examined autocorrelation by computing the correlation between the difference between a measurement and the measurement predicted by the model (Predicted measurement_n_ – measurement_n_) and this difference for the previous measurement (Predicted measurement_n-1­_ - measurement_n-1­_). To verify that the models were not dominated by individuals with large numbers of measurements, models were re-run with a random subsample of observations within each individual, such that no individual had more than the 75^th^ centile number of measurements. The coefficients from these models were very similar to those from the full model, as were residual estimates for a given individual (R≥0.9).
