# Supplementary material for: Changes in Ponderal Index and Body Mass Index across Childhood and Their Associations with Fat Mass and Cardiovascular Risk Factors at Age 15
Source: PLoS One. 2010 Dec 8;5(12):e15186. doi: 10.1371/journal.pone.0015186 (PMC2999567; doi:10.1371/journal.pone.0015186)
Supplement: File S5 — Details of multiple imputation procedure (DOCX) [file pone.0015186.s019.docx]

**Supporting File 5: Details of multiple imputation procedure**

We used switching regression in Stata as described by Royston.[5] We carried out 20 cycles of regression switching and generated 10 imputation datasets. The multivariable imputation approach creates a number of copies of the data (in this case we generated 10 copies) each of which has imputed values for those that are missing, with an appropriate level of randomness, by chained equations.[5] The main analysis results are obtained by averaging across the results from each of these 10 datasets using Rubin’s rules and the procedure takes account of uncertainty in the imputation as well as uncertainty due to random variation.[5]
