# Supplementary material for: Changes in Ponderal Index and Body Mass Index across Childhood and Their Associations with Fat Mass and Cardiovascular Risk Factors at Age 15
Source: PLoS One. 2010 Dec 8;5(12):e15186. doi: 10.1371/journal.pone.0015186 (PMC2999567; doi:10.1371/journal.pone.0015186)
Supplement: Table S1 — Description of singleton ALSPAC participants included in our analyses, and comparisons with the original cohort (DOCX) [file pone.0015186.s020.docx]

**Table S1: Description of singleton ALSPAC participants included in our analyses, and comparisons with the original cohort**

|  | **Children included in analysis (N=4601)** | **Full ALSPAC cohort (N=14,062)** | **p value** |
| --- | --- | --- | --- |
| Maternal education  Less than O-Level  O-Level  A-Level  Degree or above | 825 (18.4%)  1550 (34.6%)  1294 (28.9%)  808 (18.1%) | 3753 (30.4%)  4330 (34.7%)  2803 (22.4%)  1607 (12.9%) | <0.001 |
| Mean maternal age, years (SD) | 29.27 (4.53) | 28.00 (4.96) | <0.001 |
| Mean maternal BMI, kg/m^2^ (SD) | 22.85 (3.63) | 22.93 (3.85) | 0.21 |
| Mean birth weight, kg (SD) | 3.44 (0.52) | 3.38 (0.58) | <0.001 |
