# Supplementary material for: Changes in Ponderal Index and Body Mass Index across Childhood and Their Associations with Fat Mass and Cardiovascular Risk Factors at Age 15
Source: PLoS One. 2010 Dec 8;5(12):e15186. doi: 10.1371/journal.pone.0015186 (PMC2999567; doi:10.1371/journal.pone.0015186)
Supplement: Table S2 — Actual ponderal index (PI) and body mass index (BMI) measurements, difference between actual measurements and those predicted by the multilevel models, and estimated rates of adiposity change in all ALSPAC participants with at least 1 adiposity measure (DOCX) [file pone.0015186.s021.docx]

**Table S2: Actual ponderal index (PI) and body mass index (BMI) measurements, difference between actual measurements and those predicted by the multilevel models, and estimated rates of adiposity change in all ALSPAC participants with at least 1 adiposity measure**

|  | **Boys** |  |  |  |  | **Girls** |  |  |  |  |
| --- | --- | --- | --- | --- | --- | --- | --- | --- | --- | --- |
|  | **N** | **Mean actual measure (SD)** | **Mean difference between actual and predicted measures** | **95% level of agreement** | **Mean (SD) estimated rate of adiposity change per month** | **N** | **Mean actual measure (SD)** | **Mean difference between actual and predicted measures** | **95% level of agreement** | **Mean (SD) estimated rate of adiposity change per month** |
| ***Actual adiposity*** |  |  |  |  |  |  |  |  |  |  |
| PI at birth | 5305 | 26.03 (2.60) | 0.076 | -2.69 , 2.84 |  | 4974 | 26.36 (2.64) | 0.013 | -1.85 , 1.88 |  |
| PI 1 | 5481 | 26.40 (3.03) | -0.19 | -3.91 , 3.53 | 0.48 (0.02) | 586 | 25.43 (3.00) | -0.30 | -3.69 , 3.09 | -0.31 (0.05) |
| PI 2 | 17503 | 23.58 (3.31) | 0.040 | -2.99 , 3.06 | -0.36 (0.003) | 7247 | 26.18 (2.78) | 0.034 | -2.30 , 2.37 | 0.16 (0.02) |
| PI 3 |  |  |  |  |  | 13720 | 23.02 (3.18) | -0.015 | -2.47 , 2.44 | -0.39 (0.003) |
| BMI 1 | 8927 | 16.40 (1.44) | 0.0065 | -1.00 , 0.96 | -0.02 (0.001) | 8416 | 16.25 (1.53) | -0.017 | -1.28 , 0.92 | -0.01 (0.001) |
| BMI 2 | 1281 | 16.02 (1.74) | 0.054 | -1.69 , 1.72 | -0.03 (0.005) | 620 | 16.07 (1.72) | 0.013 | -1.27 , 1.59 | -0.05 (0.01) |
| BMI 3 | 2282 | 15.72 (1.84) | -0.0099 | -1.73 , 1.64 | -0.04 (0.02) | 2628 | 15.66 (1.93) | -0.059 | -2.12 , 2.00 | -0.01 (0.01) |
| BMI 4 | 619 | 15.78 (2.11) | 0.19 | -2.48 , 2.74 | -0.03 (0.01) | 603 | 15.92 (2.25) | 0.18 | -2.38 , 2.76 | 0.01 (0.01) |
| BMI 5 | 6451 | 16.35 (1.99) | 0.021 | -0.89 , 0.84 | 0.06 (0.001) | 4897 | 16.46 (2.13) | -0.023 | -0.73 , 0.68 | 0.07 (0.001) |
| BMI 6 | 3563 | 17.29 (2.54) | 0.0063 | -0.98 , 0.90 | 0.03 (0.002) | 4921 | 17.53 (2.68) | -0.025 | -0.85 , 0.80 | 0.04 (0.002) |

Predictions were estimated from the multilevel model. PI/BMI values were predicted at time points using individual coefficients from the multilevel model.

PI 1 : PI change between birth and 2 months for boys, birth and 1 month for girls

PI 2 : PI change between 2 and 24 months for boys, 1 and 4 months for girls

PI 3 : PI change between 4 and 24 months for girls (not applicable for boys)

BMI 1 : BMI change between 24 and 60 months for boys, 24 and 56 months for girls

BMI 2 : BMI change between 60 and 65 months for boys, 56 and 67 months for girls

BMI 3 : BMI change between 65 and 75 months for boys, 67 and 73 months for girls

BMI 4 : BMI change between 75 and 81 months for boys, 73 and 79 months for girls

BMI 5 : BMI change between 81 and 103 months for boys, 79 and 105 months for girls

BMI 6 : BMI change between 103 and 120 months for boys, 105 and 120 months for girls
