# Supplementary material for: Changes in Ponderal Index and Body Mass Index across Childhood and Their Associations with Fat Mass and Cardiovascular Risk Factors at Age 15
Source: PLoS One. 2010 Dec 8;5(12):e15186. doi: 10.1371/journal.pone.0015186 (PMC2999567; doi:10.1371/journal.pone.0015186)
Supplement: Table S3 — Adiposity trajectories from birth to ten years and their association with DXA-assessed total body fat mass at age 15 years, with multiple imputation (DOCX) [file pone.0015186.s022.docx]

**Table S3: Adiposity trajectories from birth to ten years and their association with DXA-assessed total body fat mass at age 15 years, with multiple imputation**

| Adiposity change period* | Logged DXA-assessed Fat Mass† |  |  |
| --- | --- | --- | --- |
|  | Model 1‡ | Model 2 | Model 3 |
| *Boys, N=2181* |  |  |  |
| PI at birth | 0.047 (0.004,0.090) | 0.047 (0.004,0.090) | 0.038 (-0.004,0.079) |
| PI change 0-2mt | 0.034 (-0.008,0.077) | 0.041 (-0.002,0.084) | 0.109 (0.067,0.152) |
| **PI change 2-24mt** | **-0.043 (-0.086,-0.001)** | **0.112 (-0.010,0.233)** | **0.164 (0.048,0.281)** |
| **BMI change 2-5y** | **0.242 (0.202,0.282)** | **0.235 (0.194,0.275)** | **0.172 (0.134,0.209)** |
| **BMI change 5-5.5y** | **0.270 (0.230,0.310)** | **0.665 (0.621,0.709)** | **0.558 (0.511,0.605)** |
| **BMI change 5.5-6.5y** | **-0.128 (-0.169,-0.088)** | **-0.295 (-0.335,-0.255)** | **-0.259 (-0.298,-0.220)** |
| **BMI change 6.5-7y** | **0.331 (0.292,0.370)** | **-0.556 (-0.680,-0.433)** | **-0.491 (-0.612, -0.370)** |
| BMI change 7-8.5y | 0.654 (0.623,0.686) | 0.452 (0.347,0.557) | 0.446 (0.343,0.549) |
| BMI change 8.5-10y | 0.599 (0.566,0.633) | 0.263 (0.195,0.331) | 0.232 (0.165,0.299) |
|  |  |  |  |
| *Girls, N=2420* |  |  |  |
| PI at birth | 0.098 (0.058,0.138) | 0.098 (0.058,0.138) | 0.093 (0.054,0.131) |
| **PI change 0-1m** | **0.022 (-0.021,0.064)** | **0.060 (0.016,0.105)** | **0.102 (0.058,0.146)** |
| PI change 1-4m | 0.067 (0.025,0.109) | 0.157 (0.107,0.206) | 0.218 (0.171,0.264) |
| **PI change 4-24m** | **-0.105 (-0.146,-0.064)** | **0.115 (0.033,0.198)** | **0.200 (0.126,0.273)** |
| **BMI change 2-5y** | **0.453 (0.418,0.489)** | **0.444 (0.407,0.481)** | **0.306 (0.271,0.342)** |
| **BMI change 5-5.5y** | **0.111 (0.071,0.151)** | **0.407 (0.369,0.444)** | **0.301 (0.264,0.337)** |
| **BMI change 5.5-6.5y** | **-0.058 (-0.097,-0.019)** | **-0.317 (-0.359,-0.275)** | **-0.244 (-0.286,-0.203)** |
| BMI change 6.5-7y | 0.394 (0.358,0.430) | -0.166 (-0.248,-0.084) | -0.101 (-0.178,-0.024) |
| BMI change 7-8.5y | 0.553 (0.520,0.587) | 0.275 (0.232,0.319) | 0.222 (0.180,0.263) |
| BMI change 8.5-10y | 0.487 (0.453,0.521) | -0.119 (-0.196,-0.042) | -0.063 (-0.139,0.012) |

PI = ponderal index

BMI = body mass index

SD = standard deviation

Model 1 is adjusted for age at time of measurement of the outcome only

Model 2 is adjusted for age and previous periods of PI/BMI change

Model 3 is adjusted for age, previous periods of PI/BMI change, and confounders

Model 4 is adjusted for age, previous periods of PI/BMI change, confounders, and DXA-assessed fat mass, height and height squared at age 15

**Bold text** indicates that adiposity levels tend to decrease in this period; unshaded cells indicate adiposity increases in this period

BMI change periods:

BMI change 2-5y: 24 and 60 months for boys, 24 and 56 months for girls

BMI change 5-5.5y: 60 and 65 months for boys, 56 and 67 months for girls

BMI change 5.5-6.5y: 65 and 75 months for boys, 67 and 73 months for girls

BMI change 6.5-7y: 75 and 81 months for boys, 73 and 79 months for girls

BMI change 7-8.5y: 81 and 103 months for boys, 79 and 105 months for girls

BMI change 8.5-10y: 103 and 120 months for boys, 105 and 120 months for girls

All variables are standardised, so coefficients represent the standard deviation change in the outcome that is observed with a one standard deviation increase in PI at birth or adiposity change.
