# Supplementary material for: Changes in Ponderal Index and Body Mass Index across Childhood and Their Associations with Fat Mass and Cardiovascular Risk Factors at Age 15
Source: PLoS One. 2010 Dec 8;5(12):e15186. doi: 10.1371/journal.pone.0015186 (PMC2999567; doi:10.1371/journal.pone.0015186)
Supplement: Table S4 — Adiposity trajectories from birth to ten years and their association with systolic blood pressure at age 15 years, with multiple imputation (DOCX) [file pone.0015186.s023.docx]

**Table S4: Adiposity trajectories from birth to ten years and their association with systolic blood pressure at age 15 years, with multiple imputation**

|  | Systolic blood pressure |  |  |  |
| --- | --- | --- | --- | --- |
|  | Model 1 | Model 2 | Model 3 | Model 4 |
| *Boys, N=2181* |  |  |  |  |
| PI at birth | -0.045  (-0.088,-0.002) | -0.045  (-0.088,-0.002) | -0.062  (-0.106,-0.018) | -0.062  (-0.105,-0.019) |
| PI change 0-2mt | 0.045  (0.002,0.087) | 0.039  (-0.004,0.082) | 0.049  (0.004,0.095) | 0.020  (-0.026,0.065) |
| **PI change 2-24mt** | **-0.004**  **(-0.046,0.039)** | **0.013**  **(-0.115,0.140)** | **0.053**  **(-0.076,0.182)** | **0.040**  **(-0.088,0.167)** |
| **BMI change 2-5y** | **0.046**  **(0.004,0.087)** | **0.044**  **(0.002,0.086)** | **0.014**  **(-0.028,0.056)** | **0.005**  **(-0.038,0.048)** |
| **BMI change 5-5.5y** | **0.043**  **(0.001,0.086)** | **0.113**  **(0.057,0.168)** | **0.012**  **(-0.047,0.071)** | **-0.031**  **(-0.097,0.035)** |
| **BMI change 5.5-6.5y** | **-0.064**  **(-0.105,-0.023)** | **-0.116**  **(-0.166,-0.065)** | **-0.079**  **(-0.129,-0.028)** | **-0.065**  **(-0.119,-0.012)** |
| **BMI change 6.5-7y** | **0.113**  **(0.071,0.156)** | **0.035**  **(-0.135,0.205)** | **0.086**  **(-0.086,0.258)** | **0.130**  **(-0.044,0.304)** |
| BMI change 7-8.5y | 0.126  (0.084,0.167) | 0.124  (-0.017,0.264) | 0.103  (-0.037,0.243) | 0.074  (-0.068,0.217) |
| BMI change 8.5-10y | 0.140  (0.098,0.182) | 0.063  (-0.029,0.155) | 0.026  (-0.065,0.117) | 0.023  (-0.069,0.115) |
|  |  |  |  |  |
| *Girls, N=2420* |  |  |  |  |
| PI at birth | -0.009  (-0.050,0.032) | -0.009  (-0.050,0.032) | -0.014  (-0.057,0.029) | -0.037  (-0.079,0.007) |
| **PI change 0-1m** | **0.031**  **(-0.010,0.073)** | **0.032**  **(-0.013,0.076)** | **0.036**  **(-0.011,0.083)** | **0.004**  **(-0.043,0.051)** |
| PI change 1-4m | -0.019  (-0.061,0.022) | -0.008  (-0.057,0.041) | 0.013  (-0.037,0.063) | -0.038  (-0.089,0.013) |
| **PI change 4-24m** | **-0.009**  **(-0.050,0.033)** | **0.011**  **(-0.074,0.096)** | **0.045**  **(-0.040,0.131)** | **0.005**  **(-0.080,0.090)** |
| **BMI change 2-5y** | **0.089**  **(0.048,0.129)** | **0.102**  **(0.060,0.144)** | **0.054**  **(0.010,0.099)** | **-0.008**  **(-0.056,0.039)** |
| **BMI change 5-5.5y** | **0.063**  **(0.021,0.105)** | **0.128**  **(0.080,0.176)** | **0.098**  **(0.049,0.146)** | **0.041**  **(-0.013,0.096)** |
| **BMI change 5.5-6.5y** | **-0.030**  **(-0.071,0.010)** | **-0.081**  **(-0.134,-0.028)** | **-0.062**  **(-0.116,-0.007)** | **-0.017**  **(-0.072,0.038)** |
| BMI change 6.5-7y | 0.107  (0.067,0.148) | -0.040  (-0.143,0.063) | -0.029  (-0.132,0.075) | -0.011  (-0.114,0.093) |
| BMI change 7-8.5y | 0.150  (0.110,0.189) | 0.095  (0.038,0.152) | 0.081  (0.023,0.139) | 0.043  (-0.016,0.102) |
| BMI change 8.5-10y | 0.129  (0.089,0.169) | -0.027  (-0.131,0.077) | -0.016  (-0.122,0.090) | -0.008  (-0.113,0.098) |

PI = ponderal index

BMI = body mass index

SD = standard deviation

Model 1 is adjusted for age at time of measurement of the outcome only

Model 2 is adjusted for age and previous periods of PI/BMI change

Model 3 is adjusted for age, previous periods of PI/BMI change, and confounders

Model 4 is adjusted for age, previous periods of PI/BMI change, confounders, and DXA-assessed fat mass, height and height squared at age 15

**Bold text** indicates indicate that adiposity levels tend to decrease in this period; unshaded cells indicate adiposity increases in this period

BMI change periods:

BMI change 2-5y: 24 and 60 months for boys, 24 and 56 months for girls

BMI change 5-5.5y: 60 and 65 months for boys, 56 and 67 months for girls

BMI change 5.5-6.5y: 65 and 75 months for boys, 67 and 73 months for girls

BMI change 6.5-7y: 75 and 81 months for boys, 73 and 79 months for girls

BMI change 7-8.5y: 81 and 103 months for boys, 79 and 105 months for girls

BMI change 8.5-10y: 103 and 120 months for boys, 105 and 120 months for girls

All variables are standardised, so coefficients represent the standard deviation change in the outcome that is observed with a one standard deviation increase in PI at birth or adiposity change.
