# Supplementary material for: Changes in Ponderal Index and Body Mass Index across Childhood and Their Associations with Fat Mass and Cardiovascular Risk Factors at Age 15
Source: PLoS One. 2010 Dec 8;5(12):e15186. doi: 10.1371/journal.pone.0015186 (PMC2999567; doi:10.1371/journal.pone.0015186)
Supplement: Table S5 — Adiposity trajectories from birth to ten years and their association with diastolic blood pressure at age 15 years, with multiple imputation (DOCX) [file pone.0015186.s024.docx]

**Table S5: Adiposity trajectories from birth to ten years and their association with diastolic blood pressure at age 15 years, with multiple imputation**

|  | Diastolic blood pressure |  |  |  |
| --- | --- | --- | --- | --- |
|  | Model 1 | Model 2 | Model 3 | Model 4 |
| *Boys, N=2181* |  |  |  |  |
| PI at birth | -0.042  (-0.085,0.001) | -0.042  (-0.085,0.001) | -0.026  (-0.070,0.019) | -0.021  (-0.065,0.023) |
| PI change 0-2mt | -0.001  (-0.044,0.043) | -0.006  (-0.050,0.037) | 0.034  (-0.012,0.081) | 0.023  (-0.024,0.070) |
| **PI change 2-24mt** | **0.027**  **(-0.016,0.070)** | **-0.009**  **(-0.137,0.119)** | **0.032**  **(-0.096,0.160)** | **0.040**  **(-0.088,0.167)** |
| **BMI change 2-5y** | **0.002**  **(-0.040,0.045)** | **0.005**  **(-0.038,0,048)** | **-0.001**  **(-0.044,0.042)** | **0.013**  **(-0.031,0.057)** |
| **BMI change 5-5.5y** | **0.016**  **(-0.025,0.058)** | **0.031**  **(-0.023,0.085)** | **-0.013**  **(-0.073,0.047)** | **0.038**  **(-0.028,0.105)** |
| **BMI change 5.5-6.5y** | **-0.013**  **(-0.056,0.029)** | **-0.026**  **(-0.078,0.026)** | **-0.004**  **(-0.057,0.048)** | **-0.029**  **(-0.084,0.026)** |
| **BMI change 6.5-7y** | **0.022**  **(-0.021,0.065)** | **0.014**  **(-0.154,0.181)** | **0.030**  **(-0.138,0.180)** | **0.013**  **(-0.156,0.182)** |
| BMI change 7-8.5y | 0.024  (-0.018,0.066) | 0.006  (-0.136,0.147) | -0.012  (-0.153,0.129) | 0.023  (-0.119,0.165) |
| BMI change 8.5-10y | 0.040  (-0.002,0.082) | 0.075  (-0.020,0.169) | 0.066  (-0.028,0.161) | 0.084  (-0.011,0.179) |
|  |  |  |  |  |
| *Girls, N=2420* |  |  |  |  |
| PI at birth | -0.040  (-0.082,0.002) | -0.040  (-0.082,0.002) | -0.027  (-0.070,0.016) | -0.032  (-0.076,0.011) |
| **PI change 0-1m** | **0.019**  **(-0.023,0.060)** | **0.006**  **(-0.039,0.051)** | **0.024**  **(-0.022,0.070)** | **0.006**  **(-0.040,0.053)** |
| PI change 1-4m | -0.005  (-0.046,0.036) | -0.012  (-0.061,0.037) | 0.013  (-0.038,0.064) | -0.003  (-0.055,0.048) |
| **PI change 4-24m** | **0.009**  **(-0.034,0.051)** | **-0.003**  **(-0.087,0.081)** | **0.021**  **(-0.064,0.105)** | **0.008**  **(-0.077,0.093)** |
| **BMI change 2-5y** | **-0.002**  **(-0.044,0.040)** | **0.006**  **(-0.037,0.050)** | **-0.014**  **(-0.059,0.032)** | **-0.024**  **(-0.074,0.026)** |
| **BMI change 5-5.5y** | **0.025**  **(-0.017,0.067)** | **0.035**  **(-0.014,0.083)** | **0.015**  **(-0.036,0.065)** | **0.009**  **(-0.047,0.064)** |
| **BMI change 5.5-6.5y** | **-0.009**  **(-0.050,0.031)** | **-0.011**  **(-0.066,0.044)** | **-0.002**  **(-0.057,0.054)** | **0.008**  **(-0.050,0.066)** |
| BMI change 6.5-7y | 0.002  (-0.039,0.043) | -0.076  (-0.181,0.028) | -0.077  (-0.181,0.028) | -0.070  (-0.174,0.035) |
| BMI change 7-8.5y | 0.026  (-0.015,0.068) | 0.017  (-0.042,0.075) | 0.009  (-0.049,0.068) | 0.002  (-0.058,0.063) |
| BMI change 8.5-10y | 0.021  (-0.021,0.063) | 0.024  (-0.079,0.126) | 0.039  (-0.065,0.143) | 0.043  (-0.061,0.148) |

PI = ponderal index

BMI = body mass index

SD = standard deviation

Model 1 is adjusted for age at time of measurement of the outcome only

Model 2 is adjusted for age and previous periods of PI/BMI change

Model 3 is adjusted for age, previous periods of PI/BMI change, and confounders

Model 4 is adjusted for age, previous periods of PI/BMI change, confounders, and DXA-assessed fat mass, height and height squared at age 15

**Bold text** indicates that adiposity levels tend to decrease in this period; unshaded cells indicate adiposity increases in this period

BMI change periods:

BMI change 2-5y: 24 and 60 months for boys, 24 and 56 months for girls

BMI change 5-5.5y: 60 and 65 months for boys, 56 and 67 months for girls

BMI change 5.5-6.5y: 65 and 75 months for boys, 67 and 73 months for girls

BMI change 6.5-7y: 75 and 81 months for boys, 73 and 79 months for girls

BMI change 7-8.5y: 81 and 103 months for boys, 79 and 105 months for girls

BMI change 8.5-10y: 103 and 120 months for boys, 105 and 120 months for girls

All variables are standardised, so coefficients represent the standard deviation change in the outcome that is observed with a one standard deviation increase in PI at birth or adiposity change.
