# Supplementary material for: Changes in Ponderal Index and Body Mass Index across Childhood and Their Associations with Fat Mass and Cardiovascular Risk Factors at Age 15
Source: PLoS One. 2010 Dec 8;5(12):e15186. doi: 10.1371/journal.pone.0015186 (PMC2999567; doi:10.1371/journal.pone.0015186)
Supplement: Table S6 — Adiposity trajectories from birth to ten years and their association with LDLc at age 15 years, with multiple imputation (DOCX) [file pone.0015186.s025.docx]

**Table S6: Adiposity trajectories from birth to ten years and their association with LDLc at age 15 years, with multiple imputation**

|  | LDLc |  |  |  |
| --- | --- | --- | --- | --- |
|  | Model 1 | Model 2 | Model 3 | Model 4 |
| *Boys, N=2181* |  |  |  |  |
| PI at birth | 0.025  (-0.025,0.076) | 0.025  (-0.025,0.076) | 0.013  (-0.040,0.064) | 0.004  (-0.045,0.054) |
| PI change 0-2mt | 0.027  (-0.029,0.084) | 0.031  (-0.025,0.088) | 0.027  (-0.034,0.087) | 0.020  (-0.042,0,082) |
| **PI change 2-24mt** | **-0.029**  **(-0.079,0.022)** | **0.074**  **(-0.068,0.215)** | **0.062**  **(-0.081,0.205)** | **0.031**  **(-0.109,0.171)** |
| **BMI change 2-5y** | **0.024**  **(-0.029,0.077)** | **0.020**  **(-0.033,0.073)** | **0.018**  **(-0.036,0.072)** | **-0.020**  **(-0.075,0.035)** |
| **BMI change 5-5.5y** | **0.039**  **(-0.017,0.096)** | **0.087**  **(0.012,0.162)** | **0.112**  **(0.033,0.191)** | **-0.010**  **(-0.101,0.081)** |
| **BMI change 5.5-6.5y** | **-0.017**  **(-0.067,0.033)** | **-0.033**  **(-0.092,0.027)** | **-0.046**  **(-0.107,0.014)** | **0.011**  **(-0.048,0.070)** |
| **BMI change 6.5-7y** | **0.028**  **(-0.018,0.075)** | **-0.232**  **(-0.432,-0.032)** | **-0.247**  **(-0.447,-0.046)** | **-0.163**  **(-0.359,0.034)** |
| BMI change 7-8.5y | 0.092  (0.045,0.140) | 0.063  (-0.085,0.212) | 0.068  (-0.081,0.217) | -0.021  (-0.173,0.130) |
| BMI change 8.5-10y | 0.061  (0.012,0.110) | -0.015  (-0.118,0.088) | -0.016  (-0.121,0.088) | -0.069  (-0.174,0.036) |
|  |  |  |  |  |
| *Girls, N=2420* |  |  |  |  |
| PI at birth | 0.019  (-0.034,0.072) | 0.019  (-0.034,0.072) | 0.013  (-0.041,0.067) | 0.001  (-0.054,0.055) |
| **PI change 0-1m** | **0.032**  **(-0.017,0.081)** | **0.043**  **(-0.009,0.094)** | **0.041**  **(-0.012,0.093)** | **0.040**  **(-0.015,0.096)** |
| PI change 1-4m | -0.002  (-0.059,0.054) | 0.030  (-0.047,0.106) | 0.023  (-0.055,0.101) | -0.003  (-0.084,0.078) |
| **PI change 4-24m** | **-0.038**  **(-0.093,0.017)** | **0.018**  **(-0.073,0.110)** | **0.015**  **(-0.074,0.104)** | **-0.014**  **(-0.104,0.076)** |
| **BMI change 2-5y** | **0.049**  **(-0.001,0.099)** | **0.037**  **(-0.015,0.088)** | **0.037**  **(-0.018,0.093)** | **-0.022**  **(-0.082,0.037)** |
| **BMI change 5-5.5y** | **-0.014**  **(-0.063,0.034)** | **0.013**  **(-0.047,0.073)** | **0.015**  **(-0.048,0.079)** | **-0.054**  **(-0.125,0.017)** |
| **BMI change 5.5-6.5y** | **-0.016**  **(-0.063,0.031)** | **-0.056**  **(-0.117,0.005)** | **-0.057**  **(-0.120,0.005)** | **-0.006**  **(-0.069,0.058)** |
| BMI change 6.5-7y | 0.040  (-0.008,0.088) | -0.050  (-0.168,0.068) | -0.042  (-0.163,0.080) | -0.023  (-0.144,0.097) |
| BMI change 7-8.5y | 0.060  (0.016,0.105) | 0.060  (-0.010,0.131) | 0.060  (-0.012,0.132) | 0.013  (-0.063,0.090) |
| BMI change 8.5-10y | 0.042  (-0.005,0.089) | -0.055  (-0.169,0.059) | -0.076  (-0.191,0.038) | -0.064  (-0.178,0.050) |

PI = ponderal index

BMI = body mass index

SD = standard deviation

Model 1 is adjusted for age at time of measurement of the outcome only

Model 2 is adjusted for age and previous periods of PI/BMI change

Model 3 is adjusted for age, previous periods of PI/BMI change, and confounders

Model 4 is adjusted for age, previous periods of PI/BMI change, confounders, and DXA-assessed fat mass, height and height squared at age 15

**Bold text** indicates that adiposity levels tend to decrease in this period; unshaded cells indicate adiposity increases in this period

BMI change periods:

BMI change 2-5y: 24 and 60 months for boys, 24 and 56 months for girls

BMI change 5-5.5y: 60 and 65 months for boys, 56 and 67 months for girls

BMI change 5.5-6.5y: 65 and 75 months for boys, 67 and 73 months for girls

BMI change 6.5-7y: 75 and 81 months for boys, 73 and 79 months for girls

BMI change 7-8.5y: 81 and 103 months for boys, 79 and 105 months for girls

BMI change 8.5-10y: 103 and 120 months for boys, 105 and 120 months for girls

All variables are standardised, so coefficients represent the standard deviation change in the outcome that is observed with a one standard deviation increase in PI at birth or adiposity change.
