# Supplementary material for: Changes in Ponderal Index and Body Mass Index across Childhood and Their Associations with Fat Mass and Cardiovascular Risk Factors at Age 15
Source: PLoS One. 2010 Dec 8;5(12):e15186. doi: 10.1371/journal.pone.0015186 (PMC2999567; doi:10.1371/journal.pone.0015186)
Supplement: Table S7 — Adiposity trajectories from birth to ten years and their association with HDLc at age 15 years, with multiple imputation (DOCX) [file pone.0015186.s026.docx]

**Table S7: Adiposity trajectories from birth to ten years and their association with HDLc at age 15 years, with multiple imputation**

|  | HDLc |  |  |  |
| --- | --- | --- | --- | --- |
|  | Model 1 | Model 2 | Model 3 | Model 4 |
| *Boys, N=2181* |  |  |  |  |
| PI at birth | -0.023  (-0.077,0.031) | -0.023  (-0.077,0.031) | -0.012  (-0.065,0.042) | -0.005  (-0.057,0.047) |
| PI change 0-2mt | -0.041  (-0.090,0.008) | -0.045  (-0.093,0.003) | -0.062  (-0.115,-0.008) | -0.029  (-0.083,0.025) |
| **PI change 2-24mt** | **0.040**  **(-0.011,0.090)** | **-0.053**  **(-0.213,0.108)** | **-0.063**  **(-0.222,0.096)** | **-0.030**  **(-0.187,0.127)** |
| **BMI change 2-5y** | **-0.068**  **(-0.124,-0.012)** | **-0.069**  **(-0.126,-0.011)** | **-0.045**  **(-0.104,0.013)** | **-0.016**  **(-0.074,0.043)** |
| **BMI change 5-5.5y** | **-0.070**  **(-0.132,-0.008)** | **-0.176**  **(-0.247,-0.105)** | **-0.129**  **(-0.205,-0.053)** | **-0.034**  **(-0.125,0.058)** |
| **BMI change 5.5-6.5y** | **0.019**  **(-0.032,0.070)** | **0.055**  **(-0.003,0.113)** | **0.040**  **(-0.020,0.099)** | **-0.005**  **(-0.068,0.057)** |
| **BMI change 6.5-7y** | **-0.075**  **(-0.125,-0.025)** | **0.103**  **(-0.085,0.292)** | **0.078**  **(-0.107,0.264)** | **-0.038**  **(-0.215,0.138)** |
| BMI change 7-8.5y | -0.160  (-0.210,-0.111) | -0.053  (-0.209,0.102) | -0.050  (-0.203,0.103) | 0.039  (-0.119,0.196) |
| BMI change 8.5-10y | -0.167  (-0.216,-0.118) | -0.153  (-0.260,-0.046) | -0.128  (-0.235,-0.020) | -0.090  (-0.198,0.019) |
|  |  |  |  |  |
| *Girls, N=2420* |  |  |  |  |
| PI at birth | 0.010  (-0.040,0.060) | 0.010  (-0.040,0.060) | 0.031  (-0.023,0.086) | 0.049  (-0.004,0.103) |
| **PI change 0-1m** | **-0.091**  **(-0.139,-0.043)** | **-0.099**  **(-0.152,-0.045)** | **-0.090**  **(-0.148,-0.032)** | **-0.070**  **(-0.126,-0.014)** |
| PI change 1-4m | 0.057  (0.010,0.104) | 0.016  (-0.040,0.072) | 0.027  (-0.029,0.084) | 0.073  (0.017,0.128) |
| **PI change 4-24m** | **0.033**  **(-0.017,0.084)** | **-0.042**  **(-0.130,0.046)** | **-0.055**  **(-0.143,0.033)** | **-0.014**  **(-0.102,0.073)** |
| **BMI change 2-5y** | **-0.101**  **(-0.145,-0.057)** | **-0.098**  **(-0.145,-0.051)** | **-0.061**  **(-0.112,-0.010)** | **-0.001**  **(-0.058,0.057)** |
| **BMI change 5-5.5y** | **0.024**  **(-0.021,0.070)** | **-0.031**  **(-0.082,0.020)** | **-0.001**  **(-0.054,0.053)** | **0.068**  **(-0.002,0.135)** |
| **BMI change 5.5-6.5y** | **-0.014**  **(-0.067,0.039)** | **0.056**  **(-0.015,0.128)** | **0.032**  **(-0.043,0.107)** | **-0.026**  **(-0.101,0.049)** |
| BMI change 6.5-7y | -0.058  (-0.112,-0.004) | -0.031  (-0.162,0.101) | -0.075  (-0.207,0.058) | -0.099  (-0.231,0.034) |
| BMI change 7-8.5y | -0.092  (-0.140,-0.044) | -0.059  (-0.134,0.016) | -0.038  (-0.117,0.041) | 0.016  (-0.066,0.098) |
| BMI change 8.5-10y | -0.073  (-0.121,-0.026) | 0.019  (-0.090,0.127) | 0.011  (-0.100,0.122) | -0.004  (-0.114,0.106) |

PI = ponderal index

BMI = body mass index

SD = standard deviation

Model 1 is adjusted for age at time of measurement of the outcome only

Model 2 is adjusted for age and previous periods of PI/BMI change

Model 3 is adjusted for age, previous periods of PI/BMI change, and confounders

Model 4 is adjusted for age, previous periods of PI/BMI change, confounders, and DXA-assessed fat mass, height and height squared at age 15

**Bold text** indicates that adiposity levels tend to decrease in this period; unshaded cells indicate adiposity increases in this period

BMI change periods:

BMI change 2-5y: 24 and 60 months for boys, 24 and 56 months for girls

BMI change 5-5.5y: 60 and 65 months for boys, 56 and 67 months for girls

BMI change 5.5-6.5y: 65 and 75 months for boys, 67 and 73 months for girls

BMI change 6.5-7y: 75 and 81 months for boys, 73 and 79 months for girls

BMI change 7-8.5y: 81 and 103 months for boys, 79 and 105 months for girls

BMI change 8.5-10y: 103 and 120 months for boys, 105 and 120 months for girls

All variables are standardised, so coefficients represent the standard deviation change in the outcome that is observed with a one standard deviation increase in PI at birth or adiposity change.
