# Supplementary material for: Changes in Ponderal Index and Body Mass Index across Childhood and Their Associations with Fat Mass and Cardiovascular Risk Factors at Age 15
Source: PLoS One. 2010 Dec 8;5(12):e15186. doi: 10.1371/journal.pone.0015186 (PMC2999567; doi:10.1371/journal.pone.0015186)
Supplement: Table S8 — Adiposity trajectories from birth to ten years and their association with Ln triglycerides at age 15 years, with multiple imputation (DOCX) [file pone.0015186.s027.docx]

**Table S8: Adiposity trajectories from birth to ten years and their association with Ln triglycerides at age 15 years, with multiple imputation**

|  | Ln Triglycerides |  |  |  |
| --- | --- | --- | --- | --- |
|  | Model 1 | Model 2 | Model 3 | Model 4 |
| *Boys, N=2181* |  |  |  |  |
| PI at birth | 0.009  (-0.039,0.056) | 0.009  (-0.039,0.056) | -0.009  (-0.057,0.039) | -0.018  (-0.065,0.028) |
| PI change 0-2mt | 0.044  (-0.003,0.090) | 0.046  (-0.010,0.092) | 0.034  (-0.018,0.085) | 0.009  (-0.042,0.060) |
| **PI change 2-24mt** | **-0.036**  **(-0.080,0.009)** | **0.029**  **(-0.113,0.171)** | **0.044**  **(-0.095,0.184)** | **0.006**  **(-0.130,0.142)** |
| **BMI change 2-5y** | **0.036**  **(-0.014,0.086)** | **0.037**  **(-0.014,0.088)** | **0.032**  **(-0.019,0.083)** | **-0.011**  **(-0.062,0.039)** |
| **BMI change 5-5.5y** | **0.064**  **(0.017,0.111)** | **0.132**  **(0.074,0.189)** | **0.122**  **(0.055,0.189)** | **-0.022**  **(-0.106,0.062)** |
| **BMI change 5.5-6.5y** | **0.008**  **(-0.044,0.059)** | **-0.001**  **(-0.064,0.062)** | **-0.002**  **(-0.068,0.063)** | **0.069**  **(0.001,0.138)** |
| **BMI change 6.5-7y** | **0.025**  **(-0.027,0.076)** | **-0.064**  **(-0.292,0.163)** | **-0.073**  **(-0.295,0.150)** | **0.054**  **(-0.161,0.269)** |
| BMI change 7-8.5y | 0.114  (0.068,0.161) | 0.137  (-0.022,0.296) | 0.142  (-0.016,0.299) | 0.021  (-0.143,0.184) |
| BMI change 8.5-10y | 0.124  (0.079,0.169) | 0.140  (0.033,0.241) | 0.130  (0.025,0.234) | 0.070  (-0.032,0.172) |
|  |  |  |  |  |
| *Girls, N=2420* |  |  |  |  |
| PI at birth | 0.005  (-0.043,0.052) | 0.005  (-0.043,0.052) | -0.008  (-0.058,0.041) | -0.020  (-0.068,0.029) |
| **PI change 0-1m** | **0.062**  **(0.008,0.116)** | **0.071**  **(0.013,0.129)** | **0.060**  **(0.005,0.116)** | **0.056**  **(-0.002,0.114)** |
| PI change 1-4m | -0.046  (-0.092,-0.001) | -0.016  (-0.068,0.035) | -0.025  (-0.080,0.029) | -0.050  (-0.108,0.008) |
| **PI change 4-24m** | **-0.040**  **(-0.088,0.007)** | **-0.041**  **(-0.143,0.060)** | **-0.041**  **(-0.141,0.058)** | **-0.066**  **(-0.168,0.035)** |
| **BMI change 2-5y** | **0.034**  **(-0.019,0.086)** | **0.025**  **(-0.028,0.078)** | **0.012**  **(-0.043,0.068)** | **-0.044**  **(-0.101,0.013)** |
| **BMI change 5-5.5y** | **0.024**  **(-0.032,0.080)** | **0.050**  **(-0.012,0.112)** | **0.047**  **(-0.017,0.111)** | **-0.014**  **(-0.087,0.059)** |
| **BMI change 5.5-6.5y** | **-0.028**  **(-0.083,0.026)** | **-0.062**  **(-0.134,0.011)** | **-0.060**  **(-0.134,0.013)** | **-0.017**  **(-0.087,0.054)** |
| BMI change 6.5-7y | 0.058  (0.011,0.105) | 0.002  (-0.130,0.133) | 0.019  (-0.115,0.152) | 0.034  (-0.100,0.168) |
| BMI change 7-8.5y | 0.071  (0.025,0.117) | 0.061  (-0.009,0.131) | 0.060  (-0.010,0.130) | 0.021  (-0.049,0.090) |
| BMI change 8.5-10y | 0.058  (0.011,0.104) | -0.041  (-0.166,0.084) | -0.048  (-0.172,0.076) | -0.039  (-0.163,0.085) |

PI = ponderal index

BMI = body mass index

SD = standard deviation

Model 1 is adjusted for age at time of measurement of the outcome only

Model 2 is adjusted for age and previous periods of PI/BMI change

Model 3 is adjusted for age, previous periods of PI/BMI change, and confounders

Model 4 is adjusted for age, previous periods of PI/BMI change, confounders, and DXA-assessed fat mass, height and height squared at age 15

**Bold text** indicates that adiposity levels tend to decrease in this period; unshaded cells indicate adiposity increases in this period

BMI change periods:

BMI change 2-5y: 24 and 60 months for boys, 24 and 56 months for girls

BMI change 5-5.5y: 60 and 65 months for boys, 56 and 67 months for girls

BMI change 5.5-6.5y: 65 and 75 months for boys, 67 and 73 months for girls

BMI change 6.5-7y: 75 and 81 months for boys, 73 and 79 months for girls

BMI change 7-8.5y: 81 and 103 months for boys, 79 and 105 months for girls

BMI change 8.5-10y: 103 and 120 months for boys, 105 and 120 months for girls

All variables are standardised, so coefficients represent the standard deviation change in the outcome that is observed with a one standard deviation increase in PI at birth or adiposity change.
