# Supplementary material for: Changes in Ponderal Index and Body Mass Index across Childhood and Their Associations with Fat Mass and Cardiovascular Risk Factors at Age 15
Source: PLoS One. 2010 Dec 8;5(12):e15186. doi: 10.1371/journal.pone.0015186 (PMC2999567; doi:10.1371/journal.pone.0015186)
Supplement: Table S9 — Adiposity trajectories from birth to ten years and their association with Ln CRP at age 15 years, with multiple imputation (DOCX) [file pone.0015186.s028.docx]

**Table S9: Adiposity trajectories from birth to ten years and their association with Ln CRP at age 15 years, with multiple imputation**

|  | Ln CRP |  |  |  |
| --- | --- | --- | --- | --- |
|  | Model 1 | Model 2 | Model 3 | Model 4 |
| *Boys, N=2181* |  |  |  |  |
| PI at birth | -0.011  (-0.066,0.044) | -0.011  (-0.066,0.044) | -0.026  (-0.080,0.029) | -0.038  (-0.91,0.015) |
| PI change 0-2mt | 0.040  (-0.011,0.091) | 0.039  (-0.013,0.091) | 0.042  (-0.011,0.095) | 0.011  (-0.043,0.065) |
| **PI change 2-24mt** | **-0.026**  **(-0.082,0.030)** | **-0.021**  **(-0.179,0.138)** | **-0.028**  **(-0.194,0.139)** | **-0.080**  **(-0.241,0.082)** |
| **BMI change 2-5y** | **0.056**  **(0.005,0.108)** | **0.052**  **(-0.004,0.108)** | **0.025**  **(-0.030,0.081)** | **-0.032**  **(-0.084,0.021)** |
| **BMI change 5-5.5y** | **0.122**  **(0.074,0.169)** | **0.248**  **(0.184,0.312)** | **0.211**  **(0.139,0.283)** | **0.034**  **(-0.053,0.121)** |
| **BMI change 5.5-6.5y** | **-0.065**  **(-0.114,-0.016)** | **-0.107**  **(-0.175,-0.039)** | **-0.096**  **(-0.163,-0.028)** | **-0.015**  **(-0.079,0.048)** |
| **BMI change 6.5-7y** | **0.127**  **(0.076,0.179)** | **-0.141**  **(-0.350,0.067)** | **-0.119**  **(-0.319,0.082)** | **0.024**  **(-0.177,0.226)** |
| BMI change 7-8.5y | 0.123  (0.151,0.274) | -0.001  (-0.188,0.185) | -0.009  (-0.190,0.171) | -0.151  (-0.326,0.024) |
| BMI change 8.5-10y | 0.217  (0.161,0.273) | 0.181  (0.079,0.284) | 0.167  (0.061,0.273) | 0.096  (-0.008,0.199) |
|  |  |  |  |  |
| *Girls, N=2420* |  |  |  |  |
| PI at birth | 0.029  (-0.022,0.079) | 0.029  (-0.022,0.079) | 0.014  (-0.037,0.064) | -0.015  (-0.065,0.034) |
| **PI change 0-1m** | **0.031**  **(-0.014,0.075)** | **0.045**  **(-0.005,0.094)** | **0.040**  **(-0.013,0.093)** | **0.022**  **(-0.027,0.072)** |
| PI change 1-4m | 0.035  (-0.023,0.093) | 0.086  (0.019,0.153) | 0.089  (0.014,0.163) | 0.029  (-0.045,0.104) |
| **PI change 4-24m** | **-0.064**  **(-0.110,-0.018)** | **0.044**  **(-0.058,0.145)** | **0.063**  **(-0.043,0.168)** | **0.007**  **(-0.097,0.112)** |
| **BMI change 2-5y** | **0.180**  **(0.120,0.241)** | **0.172**  **(0.108,0.237)** | **0.127**  **(0.061,0.193)** | **0.030**  **(-0.034,0.094)** |
| **BMI change 5-5.5y** | **0.030**  **(-0.018,0.077)** | **0.146**  **(0.095,0198)** | **0.116**  **(0.062,0.170)** | **0.016**  **(-0.043,0.076)** |
| **BMI change 5.5-6.5y** | **-0.012**  **(-0.062,0.037)** | **-0.109**  **(-0.169,-0.049)** | **-0.089**  **(-0.151,-0.026)** | **-0.016**  **(-0.083,0.051)** |
| BMI change 6.5-7y | 0.145  (0.101,0.189) | -0.037  (-0.154,0.080) | -0.014  (-0.130,0.103) | 0.011  (-0.106,0.128) |
| BMI change 7-8.5y | 0.192  (0.138,0.245) | 0.074  (0.008,0.141) | 0.057  (-0.010,0.123) | -0.011  (-0.077,0.054) |
| BMI change 8.5-10y | 0.176  (0.128,0.225) | -0.052  (-0.202,0.098) | -0.042  (-0.192,0.109) | -0.028  (-0.177,0.122) |

PI = ponderal index

BMI = body mass index

SD = standard deviation

Model 1 is adjusted for age at time of measurement of the outcome only

Model 2 is adjusted for age and previous periods of PI/BMI change

Model 3 is adjusted for age, previous periods of PI/BMI change, and confounders

Model 4 is adjusted for age, previous periods of PI/BMI change, confounders, and DXA-assessed fat mass, height and height squared at age 15

**Bold text** indicates that adiposity levels tend to decrease in this period; unshaded cells indicate adiposity increases in this period

BMI change periods:

BMI change 2-5y: 24 and 60 months for boys, 24 and 56 months for girls

BMI change 5-5.5y: 60 and 65 months for boys, 56 and 67 months for girls

BMI change 5.5-6.5y: 65 and 75 months for boys, 67 and 73 months for girls

BMI change 6.5-7y: 75 and 81 months for boys, 73 and 79 months for girls

BMI change 7-8.5y: 81 and 103 months for boys, 79 and 105 months for girls

BMI change 8.5-10y: 103 and 120 months for boys, 105 and 120 months for girls

All variables are standardised, so coefficients represent the standard deviation change in the outcome that is observed with a one standard deviation increase in PI at birth or adiposity change.
