# Supplementary material for: Changes in Ponderal Index and Body Mass Index across Childhood and Their Associations with Fat Mass and Cardiovascular Risk Factors at Age 15
Source: PLoS One. 2010 Dec 8;5(12):e15186. doi: 10.1371/journal.pone.0015186 (PMC2999567; doi:10.1371/journal.pone.0015186)
Supplement: Table S10 — Adiposity trajectories from birth to ten years and their association with Ln insulin at age 15 years, with multiple imputation (DOCX) [file pone.0015186.s029.docx]

**Table S10: Adiposity trajectories from birth to ten years and their association with Ln insulin at age 15 years, with multiple imputation**

|  | Ln insulin |  |  |  |
| --- | --- | --- | --- | --- |
|  | Model 1 | Model 2 | Model 3 | Model 4 |
| *Boys, N=2181* |  |  |  |  |
| *Boys, N=2181* | 0.002  (-0.051,0.054) | 0.002  (-0.051,0.054) | -0.018  (-0.071,0.035) | -0.034  (-0.084,0.016) |
| PI at birth | -0.034  (-0.087,0.018) | -0.035  (-0.089,0.020) | -0.040  (-0.098,0.017) | -0.092  (-0.146,0.038) |
| **PI change 0-2mt** | **0.021**  **(-0.037,0.078)** | **-0.040**  **(-0.186,0.107)** | **-0.055**  **(-0.201,0.092)** | **-0.125**  **(-0.270,0.020)** |
| **PI change 2-24mt** | **0.078**  **(0.026,0.131)** | **0.079**  **(0.025,0.133)** | **0.053**  **(-0.001,0.106)** | **-0.022**  **(-0.073,0.030)** |
| **BMI change 2-5y** | **0.117**  **(0.068,0.165)** | **0.257**  **(0.198,0.315)** | **0.226**  **(0.158,0.294)** | **-0.023**  **(-0.096,0.050)** |
| **BMI change 5-5.5y** | **-0.036**  **(-0.100,0.027)** | **-0.101**  **(-0.166,-0.036)** | **-0.089**  **(-0.154,-0.024)** | **0.028**  **(-0.035,0.091)** |
| **BMI change 5.5-6.5y** | **0.111**  **(0.054,0.168)** | **-0.184**  **(-0.360,-0.008)** | **-0.156**  **(-0.333,0.021)** | **0.070**  **(-0.104,0.244)** |
| BMI change 6.5-7y | 0.252  (0.205,0.299) | 0.352  (0.182,0.522) | 0.351  (0.184,0.519) | 0.151  (-0.013,0.316) |
| BMI change 7-8.5y | 0.238  (0.194,0.283) | 0.104  (0.003,0.207) | 0.090  (-0.015,0.195) | -0.009  (-0.115,0.096) |
| BMI change 8.5-10y |  |  |  |  |
|  |  |  |  |  |
| *Girls, N=2420* | 0.022  (-0.035,0.078) | 0.022  (-0.035,0.078) | -0.002  (-0.061,0.056) | -0.030  (-0.087,0.027) |
| **PI at birth** | **0.006**  **(-0.048,0.060)** | **0.015**  **(-0.037,0.068)** | **0.002**  **(-0.054,0.058)** | **-0.034**  **(-0.090,0.022)** |
| PI change 0-1m | 0.002  (-0.046,0.051) | 0.018  (-0.041,0.078) | 0.007  (-0.057,0.072) | -0.063  (-0.126,-0.001) |
| **PI change 1-4m** | **-0.012**  **(-0.062,0.038)** | **0.040**  **(-0.055,0.135)** | **0.063**  **(-0.034,0.159)** | **0.002**  **(-0.096,0.100)** |
| **PI change 4-24m** | **0.055**  **(0.007,0.103)** | **0.042**  **(-0.006,0.090)** | **-0.007**  **(-0.061,0.048)** | **-0.112**  **(-0.174,-0.050)** |
| **BMI change 2-5y** | **0.079**  **(0.013,0.146)** | **0.141**  **(0.064,0.217)** | **0.106**  **(0.025,0.186)** | **0.004**  **(-0.075,0.082)** |
| **BMI change 5-5.5y** | **-0.060**  **(-0.110,-0.010)** | **-0.089**  **(-0.154,-0.023)** | **-0.068**  **(-0.138,0.001)** | **0.017**  **(-0.049,0.084)** |
| BMI change 5.5-6.5y | 0.114  (0.066,0.163) | -0.017  (-0.133,0.098) | 0.019  (-0.099,0.138) | 0.056  (-0.065,0.176) |
| BMI change 6.5-7y | 0.169  (0.121,0.217) | 0.158  (0.082,0.233) | 0.137  (0.061,0.214) | 0.063  (-0.012,0.128) |
| BMI change 7-8.5y | 0.137  (0.093,0.181) | 0.060  (-0.046,0.166) | 0.065  (-0.041,0.170) | 0.086  (-0.015,0.187) |
| BMI change 8.5-10y |  |  |  |  |

PI = ponderal index

BMI = body mass index

SD = standard deviation

Model 1 is adjusted for age at time of measurement of the outcome only

Model 2 is adjusted for age and previous periods of PI/BMI change

Model 3 is adjusted for age, previous periods of PI/BMI change, and confounders

Model 4 is adjusted for age, previous periods of PI/BMI change, confounders, and DXA-assessed fat mass, height and height squared at age 15

**Bold text** indicates that adiposity levels tend to decrease in this period; unshaded cells indicate adiposity increases in this period

BMI change periods:

BMI change 2-5y: 24 and 60 months for boys, 24 and 56 months for girls

BMI change 5-5.5y: 60 and 65 months for boys, 56 and 67 months for girls

BMI change 5.5-6.5y: 65 and 75 months for boys, 67 and 73 months for girls

BMI change 6.5-7y: 75 and 81 months for boys, 73 and 79 months for girls

BMI change 7-8.5y: 81 and 103 months for boys, 79 and 105 months for girls

BMI change 8.5-10y: 103 and 120 months for boys, 105 and 120 months for girls

All variables are standardised, so coefficients represent the standard deviation change in the outcome that is observed with a one standard deviation increase in PI at birth or adiposity change.
