# Supplementary material for: Changes in Ponderal Index and Body Mass Index across Childhood and Their Associations with Fat Mass and Cardiovascular Risk Factors at Age 15
Source: PLoS One. 2010 Dec 8;5(12):e15186. doi: 10.1371/journal.pone.0015186 (PMC2999567; doi:10.1371/journal.pone.0015186)
Supplement: Table S11 — Adiposity trajectories from birth to ten years and their association with glucose at age 15 years, with multiple imputation (DOCX) [file pone.0015186.s030.docx]

**Table S11: Adiposity trajectories from birth to ten years and their association with glucose at age 15 years, with multiple imputation**

|  | **Glucose** |  |  |  |
| --- | --- | --- | --- | --- |
|  | **Model 1** | **Model 2** | **Model 3** | **Model 4** |
| ***Boys, N=2181*** |  |  |  |  |
| **PI at birth** | -0.013  (-0.061,0.035) | -0.013  (-0.061,0.035) | -0.028  (-0.078,0.022) | -0.033  (-0.083,0.017) |
| **PI change 0-2mt** | -0.029  (-0.078,0.021) | -0.031  (-0.082,0.020) | -0.038  (-0.094,0.019) | -0.052  (-0.109,0.004) |
| **PI change 2-24mt** | 0.028  (-0.027,0.083) | -0.025  (-0.169,0.120) | -0.015  (-0.0159,0.130) | -0.037  (-0.179,0.105) |
| **BMI change 2-5y** | 0.011  (-0.045,0.068) | 0.009  (-0.049,0.067) | -0.001  (-0.061,0.060) | -0.026  (-0.088,0.037) |
| **BMI change 5-5.5y** | 0.041  (-0.013,0.096) | 0.060  (-0.005,0.124) | 0.400  (-0.035,0.114) | -0.055  (-0.141,0.031) |
| **BMI change 5.5-6.5y** | 0.010  (-0.043,0.063) | 0.005  (-0.049,0.059) | 0.015  (-0.040,0.070) | -0.063  (0.006,0.120) |
| **BMI change 6.5-7y** | 0.002  (-0.044,0.047) | -0.097  (-0.274,0.080) | -0.063  (-0.241,0,115) | 0.019  (-0.162,0.199) |
| **BMI change 7-8.5y** | 0.062  (0.016,0.108) | 0.103  (-0.056,0.262) | 0.103  (-0.055,0.261) | 0.023  (-0.142,0.189) |
| **BMI change 8.5-10y** | 0.065  (0.018,0.112) | 0.080  (-0.020,0.180) | 0.068  (-0.032,0.168) | 0.028  (-0.076,0.133) |
|  |  |  |  |  |
| ***Girls, N=2420*** |  |  |  |  |
| **PI at birth** | 0.028  (-0.020,0.077) | 0.028  (-0.020,0.077) | 0.010  (-0.042,0.062) | 0.007  (-0.045,0.058) |
| **PI change 0-1m** | 0.027  (-0.027,0.081) | 0.041  (-0.013,0.095) | 0.032  (-0.019,0.083) | 0.028  (-0.023,0.078) |
| **PI change 1-4m** | -0.031  (-0.083,0.021) | -0.009  (-0.080,0.061) | -0.015  (-0.083,0.052) | -0.024  (-0.090,0.042) |
| **PI change 4-24m** | -0.015  (-0.068,0,039) | 0.029  (-0.061,0.119) | 0.029  (-0.061,0.119) | 0.026  (-0.066,0.119) |
| **BMI change 2-5y** | 0.005  (-0.043,0,053) | -0.015  (-0.064,0.034) | -0.019  (-0.073,0.034) | -0.031  (-0.089,0.027) |
| **BMI change 5-5.5y** | -0.008  (-0.075,0.059) | -0.003  (-0.078,0.071) | -0.008  (-0.084,0.068) | -0.023  (-0.109,0.063) |
| **BMI change 5.5-6.5y** | -0.012  (-0.065,0.041) | -0.023  (-0.094,0.048) | -0.020  (-0.090,0.050) | -0.009  (-0.079,0.061) |
| **BMI change 6.5-7y** | 0.016  (-0.033,0.066) | 0.025  (-0.086,0.137) | 0.035  (-0.077,0.147) | 0.039  (-0.074,0.151) |
| **BMI change 7-8.5y** | 0.021  (-0.024,0.067) | 0.051  (-0.037,0.138) | 0.053  (-0.037,0.142) | 0.045  (-0.050,0.139) |
| **BMI change 8.5-10y** | 0.015  (-0.035,0.064) | 0.016  (-0.095,0.127) | 0.011  (-0.099,0.120) | 0.011  (-0.099,0.121) |

PI = ponderal index

BMI = body mass index

SD = standard deviation

Model 1 is adjusted for age at time of measurement of the outcome only

Model 2 is adjusted for age and previous periods of PI/BMI change

Model 3 is adjusted for age, previous periods of PI/BMI change, and confounders

Model 4 is adjusted for age, previous periods of PI/BMI change, confounders, and DXA-assessed fat mass, height and height squared at age 15

Shaded cells indicate that adiposity levels tend to decrease in this period; unshaded cells indicate adiposity increases in this period

BMI change periods:

BMI change 2-5y: 24 and 60 months for boys, 24 and 56 months for girls

BMI change 5-5.5y: 60 and 65 months for boys, 56 and 67 months for girls

BMI change 5.5-6.5y: 65 and 75 months for boys, 67 and 73 months for girls

BMI change 6.5-7y: 75 and 81 months for boys, 73 and 79 months for girls

BMI change 7-8.5y: 81 and 103 months for boys, 79 and 105 months for girls

BMI change 8.5-10y: 103 and 120 months for boys, 105 and 120 months for girls

All variables are standardised, so coefficients represent the standard deviation change in the outcome that is observed with a one standard deviation increase in PI at birth or adiposity change.
