# Supplementary material for: Changes in Ponderal Index and Body Mass Index across Childhood and Their Associations with Fat Mass and Cardiovascular Risk Factors at Age 15
Source: PLoS One. 2010 Dec 8;5(12):e15186. doi: 10.1371/journal.pone.0015186 (PMC2999567; doi:10.1371/journal.pone.0015186)
Supplement: Table S12 — Associations between cardiovascular risk factors and DXA-assessed fat mass, both measured at age 15 years (DOCX) [file pone.0015186.s031.docx]

**Table S12: Associations between cardiovascular risk factors and DXA-assessed fat mass, both measured at age 15 years**

Linear regression coefficients, representing the change in cardiovascular risk factor associated with a one percent increase in DXA-assessed fat mass, adjusted for age at clinic attendance and gender

| **Cardiovascular risk factor** | **Association with fat mass and 95% confidence interval** |
| --- | --- |
| Systolic blood pressure (mmHg) | 3.56 (2.99 to 4.12) |
| Diastolic blood pressure (mmHg) | 0.30 (-0.18 to 0.78) |
| Fasting LDL cholesterol (mmol/l) | 0.15 (0.12 to 0.19) |
| Fasting HDL cholesterol (mmol/l) | -0.13 (-0.15 to -0.11) |
| Fasting triglycerides (mmol/l) | 0.14 (0.12 to 0.17) |
| Fasting glucose (mmol/l) | 0.07 (0.04 to 0.09) |
| Fasting insulin (IU/l) | 3.84 (3.51 to 4.17) |
| C-reactive protein (mg/l) | 0.64 (0.38 to 0.90) |
